# Systematic Pharmacogenomics Analysis of a Malay Whole Genome: Proof of Concept for Personalized Medicine

**DOI:** 10.1371/journal.pone.0071554

**Published:** 2013-08-23

**Authors:** Mohd Zaki Salleh, Lay Kek Teh, Lian Shien Lee, Rose Iszati Ismet, Ashok Patowary, Kandarp Joshi, Ayesha Pasha, Azni Zain Ahmed, Roziah Mohd Janor, Ahmad Sazali Hamzah, Aishah Adam, Khalid Yusoff, Boon Peng Hoh, Fazleen Haslinda Mohd Hatta, Mohamad Izwan Ismail, Vinod Scaria, Sridhar Sivasubbu

**Affiliations:** 1 Integrative Pharmacogenomics Institute (iPROMISE), Universiti Teknologi MARA (UiTM) Malaysia, Puncak Alam, Selangor, Malaysia; 2 GN Ramachandran Knowledge Center for Genome Informatics, CSIR Institute of Genomics and Integrative Biology (CSIR-IGIB), Delhi, India; 3 Genomics and Molecular Medicine, CSIR Institute of Genomics and Integrative Biology (CSIR-IGIB), Delhi, India; 4 Institute of Science, Universiti Teknologi MARA (UiTM) Malaysia, Shah Alam, Selangor, Malaysia; 5 Faculty of Computer and Mathematical Science, Universiti Teknologi MARA (UiTM) Malaysia, Shah Alam, Selangor, Malaysia; 6 Faculty of Pharmacy, Universiti Teknologi MARA (UiTM) Malaysia, Puncak Alam, Selangor, Malaysia; 7 Faculty of Medicine, Universiti Teknologi MARA (UiTM) Malaysia, Sg Buloh, Selangor, Malaysia; 8 Insitute of Medical Molecular Biotechnology (IMMB), Faculty of Medicine, Universiti Teknologi MARA (UiTM) Malaysia, Sg Buloh, Selangor, Malaysia; Tel Aviv University, Israel

## Abstract

**Background:**

With a higher throughput and lower cost in sequencing, second generation sequencing technology has immense potential for translation into clinical practice and in the realization of pharmacogenomics based patient care. The systematic analysis of whole genome sequences to assess patient to patient variability in pharmacokinetics and pharmacodynamics responses towards drugs would be the next step in future medicine in line with the vision of personalizing medicine.

**Methods:**

Genomic DNA obtained from a 55 years old, self-declared healthy, anonymous male of Malay descent was sequenced. The subject's mother died of lung cancer and the father had a history of schizophrenia and deceased at the age of 65 years old. A systematic, intuitive computational workflow/pipeline integrating custom algorithm in tandem with large datasets of variant annotations and gene functions for genetic variations with pharmacogenomics impact was developed. A comprehensive pathway map of drug transport, metabolism and action was used as a template to map non-synonymous variations with potential functional consequences.

**Principal Findings:**

Over 3 million known variations and 100,898 novel variations in the Malay genome were identified. Further in-depth pharmacogenetics analysis revealed a total of 607 unique variants in 563 proteins, with the eventual identification of 4 drug transport genes, 2 drug metabolizing enzyme genes and 33 target genes harboring deleterious SNVs involved in pharmacological pathways, which could have a potential role in clinical settings.

**Conclusions:**

The current study successfully unravels the potential of personal genome sequencing in understanding the functionally relevant variations with potential influence on drug transport, metabolism and differential therapeutic outcomes. These will be essential for realizing personalized medicine through the use of comprehensive computational pipeline for systematic data mining and analysis.

## Introduction

Recent advances in nucleotide sequencing technology have made it possible to understand personal genomes at a scale and cost not possible before [1]. These changes in the throughput of genome sequencing will have a consequential impact on the quality of healthcare and genomic services available to individuals and patients alike [2], [3]. Second generation sequencing technology has also enabled researchers to study the genomes of multiple individuals in a population as well as provide deep insights into the patterns of human migration and natural processes like selection [4]. A number of personal genomes have become publicly available in the recent past [5], [6], [7], [8], [9], [10], [11], [12] with several more genomes becoming increasingly available in private databases. Global initiatives, such as the 1000 Genomes project [13], have spearheaded the creation of a comprehensive catalogue of the genetic variations found in humans. Genomes as variegated/diverse as those found in the populations of India [8], [14] and China [11], to the homogeneous populations of Korea [5], [7] and Japan [6], along with those from smaller populations like Sri Lanka [15] have been compiled. Apart from these, there have also been concerted efforts to share and organize personal genome datasets to enable population level analysis, which includes the HUGO Pan-Asian Population Genomics Initiative [http://papgi.org ]. These efforts would have far reaching implications in the understanding of phenotype-genotype correlations on one hand while at the same time providing baseline data on disease predispositions and pharmacogenomics associations, which would provide a starting point for the population level modeling of selection and pharmacogenomics evaluations.

The second generation sequencing technology has immense potential for translation into clinical practice and in the realization of pharmacogenomics based patient care. The systematic analysis of whole genome sequences to assess patient to patient variability in pharmacokinetics and pharmacodynamics responses towards drugs would be the next step in future medicine in line with the vision of personalizing medicine [16]. Considering that the pharmacokinetics and pharmacodynamics of most drugs have been studied, the information on their pathways and targets so garnered would provide a template for the genome scale evaluation of the impact of genetic variations. Variability in drug responses were known to be influenced by both the intrinsic and extrinsic factors with genetic factors accounting for 20–95% of the patient variability [16], [17]. The fate of a drug in a biological system is largely determined by its Absorption, Distribution, Metabolism, and Excretion (ADME) properties, and these properties have been shown to be intricately linked to the genetic makeup of an individual. For example, genetic polymorphisms in the drug metabolizing enzymes such as Cytochrome P450s (CYP450s) have been shown to influence the fate and rate of drug metabolism [16] and even patient's predisposition to adverse drug reactions [18], [19]. Apart from predicting predilection to adverse drug reactions [20], the dose of drugs can also be adjusted based on the genetic profile of an individual. Genotype-phenotype association studies for particular phenotypic trait(s) have largely been conducted using a candidate gene approach or recently a Genome-Wide Association study approach. The latter extensively relies on common variations and miss out on novel variants, which may have high penetrance and hence exert functional effects. Both these approaches were based on a phenotype to genotype association or rather in simplistic sense, identifying a set of genetic variants occurring predominantly in a phenotypic cohort compared to controls. The advent of genome sequencing has opened up a new possibility that in-depth analysis of genotypes which could potentially predict traits which could be further evaluated and confirmed, thus offering a new opportunity towards understanding the phenotypic consequences of genetic variations. This would have great ramifications in an individual patient's therapeutics since deleterious phenotypes caused by rare variants are not regularly detected by the popular microarray based systems largely used for genome-wide association studies which are heavily reliant on common polymorphisms. Large databases curating pharmacogenomics information such as PharmGKB and the OpenPGx Consortium [www.openpgx.org] and other datasets including those available from the NIH GWAS Collection [20], [21] of pharmacogenomics traits have made it possible to computationally analyze personal genomes for potential translation of pharmacogenomics into clinical practice. This application of genomic technology in clinical settings would be further benefited by the development of faster and advanced computational algorithms and workflows, which would enable the systematic and efficient mining of genetic variations and their functional correlates. Furthermore, the availability of these computational tools including software codes in the public domain under open licenses would enhance their large scale adoption into clinical settings.

Malays comprise of ethnic groups of Austronesian origin who speak Malayo-Polynesian language and inhabit the Malay Peninsula. Malay has a vivid history with anecdotes of their high mobility and migration. Earlier population genetic analysis of the individuals from the Malay Peninsula has revealed significant structures, some of them having shared affinities with the Indonesian populations [22]. The Malays are one of the major populations in Southeast Asia region, in particular the Malay Archipelago. This ethnic group has a unique history of migration and gene pool. The recent investigation with 50,000 autosomal SNP [23] suggested that their gene pool is the admixed of several different populations comprising proto-Malays, Indian, Thai, Arab, Chinese and Javanese. To understand better the Malay characters in relation to genes and environment as well as migration and evolution, sequencing of the whole genome of Malays is timely and needed. The sequencing of the Malay genome will therefore provide a starting point towards understanding the genome of a representative of a large ethnically diverse population including the Malays in Malaysia, Singapore, Indonesia, Philippines and more.

In the present analysis, we describe the use of a systematic, intuitive computational workflow/pipeline integrating algorithm in tandem with large datasets of variant annotations and gene functions to mine for genetic variations, which may have potential effects on the pharmacokinetics and pharmacodynamics of drugs. The pipeline was further validated of its applicability and clinical utility using the computational workflow/pipeline of the genome of an anonymous individual of Malay descent. We also mapped the variations and genes involved in the analysis on a comprehensive pathway map created through open collaboration. We hope that our study will provide the bioinformatics analysis pipeline and be the starting point for the systematic evaluation and analysis of personal genomes with its eventual application in planning therapeutic options.

## Materials and Methods

### Subject Selection and Karyotyping

The whole genome sequencing protocol has been approved by the local Research Ethics Committee Universiti Teknologi MARA Malaysia and written informed consent for the use of data for publication and further medical related researches has been obtained from the volunteer.

The whole blood was collected from a 55 years old, self-declared healthy, anonymous male of Malay descent whose mother died of lung cancer and the father had a history of Schizophrenia and deceased at the age of 65 years old. In November 2011, following the completion of the whole genome sequencing of this individual, an ultrasound examination of the abdomen in consequence of complaints of frequent urination revealed an enlarged prostate (6.0×4.5×4.3 cm), with the central portion of the organ extending to the bladder. An examination of the prostate specific antigen level showed values that were higher than normal (6.6 ng/mL; normal <3.5). Other routine biochemical parameters were also analyzed and elevated levels of the Rheumatoid factor (16.8IU/ml; normal <15) and HDL Cholesterol (1.15 mmol/l; normal>1.42) were observed. In addition, the subject also had episodes of high blood glucose which, following a reduction in sugar intake dropped to a fasting blood glucose level of 4.48 mmol/l (normal range: 3.9–6.1). Karyotyping was carried out to conform that the volunteer is free off any large structural aberrations (Figure S1).

### DNA Isolation and Library Preparation

Isolation and purification of the DNA extracted from the whole blood was carried out using the Wizard^®^ SV Genomic DNA Purification System kit (Promega Corporation, Wisconsin, USA) as per the manufacturer's instructions. The resultant DNA pellet was reconstituted in 250 μl of TE buffer and the libraries were prepared according to the manufacturer's instructions (Illumina Inc., California, USA). Briefly, 10 µg of the gDNA in 100 μl TE buffer was fragmented by sonication (Sono Swiss, Ramsen, Sweden) at high power for 9 minutes. Fragmented gDNA were blunt ended using T4 DNA ligase and Klenow enzyme. An ‘A’ base was then added to the ends of the double-stranded DNA using Klenowexo (3′ to 5′ exo minus). The paired end (PE) adaptor (Illumina Inc., California, USA) with a single ‘T’ base overhang at the 3′ end was ligated to the above products. The PE adaptor ligated products were then separated on a 2% agarose gel. DNA fragments resolved at positions approximate to 400 bp to 500 bp were excised. Finally, size-selected DNA fragments were enriched by PCR with PE primers 1.1 and 2.1 (Illumina Inc., California, USA). The concentration of the libraries was measured by both qPCR (BioRad, California, USA) and Qubit IT (Invitrogen, Life Technologies Corporation, NY, USA). Finally, the libraries were validated by Bioanalyzer 2100 (Agilent Technologies, California, USA).

### Whole Genome Sequencing and Reference Mapping

Whole genome sequencing of the DNA from this individual was carried out using the Genome Analyzer IIx (Illumina, San Diego, California, USA) according to the manufacturer's instructions. Data was generated from multiple libraries of 100 base pair paired-end (PE) runs. Paired-end reads were mapped to the Human Reference Genome build hg19 using a quality aware short read aligner i.e. the Burrows-Wheeler Aligner (BWA) software [24]. The reference genome sequence was retrieved from the UCSC Genome Bioinformatics website [25]. BWA was run using default parameters described in the manual. The alignments were further analyzed using SAMtools software. The variations were called using the SAMtools software [26] for which the Sequence Alignment/Map (SAM) format [26] files were initially converted to the indexed and BAM sorted files following which the *mpileup* option was used and the variants exported in the standard VCF format [27]. Detailed parameters used in variation calling are summarized in data S1. In addition, the datasets, workflow and scripts are also made available for future applications and development through the OpenPGx consortium [www.openpgx.org]. The genomic variations are available at the Malay Genome Browser hyperlinked at http://promisegenome.uitm.edu.my/[http://promisegenome.uitm.edu.my/cgi-bin/gbrowse/hgmalay/ or http:// 58.26.181.172/cgi-bin/gbrowse/hgmalay using internet explorer] and integrated with data from other Asian Genomes for easy comparison and integrative analysis at the Asian Genome Browser hyperlinked at [http://genome.igib.res.in/cgi-bin/gbrowse/AsianGenome/]. Researchers interested in sharing the BAM files for researches and not for commercial purposes are invited to write in to the corresponding author. The study accession number (SRA) is PRJEB4210.

### Workflow for Analysis of Genomic Variations

**Figure 1:**
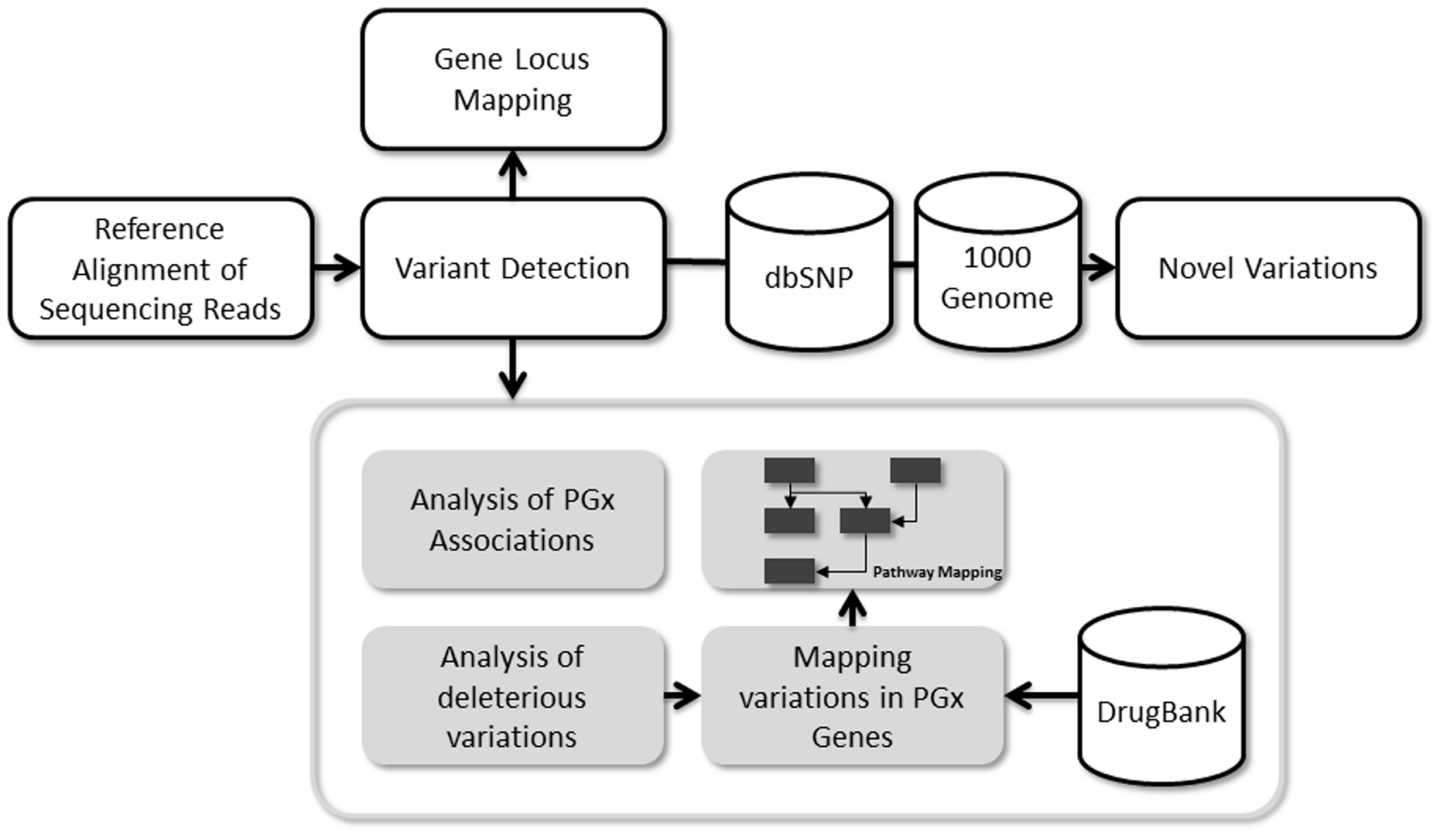
Summary of the analysis workflow for the Malaysian genome.

We created a custom workflow for the analysis of the genome. This included calling variations from the alignments, comparison with other variant databases including dbSNP [28], database of genetic variants [29] and those from the 1000 Genomes Consortium [13]. The workflow further included the mapping and comparison of markers associated with pharmacogenomics traits and the analysis of potential deleterious variations across genes responsible for pharmacokinetic and pharmacodynamics variations. The major components of the analysis workflow are summarized in Figure 1.

### Comparison and Mapping of Genomic Variations across Variant Annotation Datasets

The SNVs were called using the SAMtools software using parameters as described in data S1 and analyzed by integrating the publicly available datasets from dbSNP [28] and 1000 Genomes Consortium [13]. The Single Nucleotide Variations (SNVs) were further analyzed with respect to their relation to genes using ANNOVAR [30], a publicly available suite of software for variant analysis. Datasets of gene annotation were integrated from the UCSC Genome Resource, the Database of Genomic Variants (DGV) [29], the NHGRI GWAS Catalog [21] and the 1000 Genomes project [13]. Other datasets used in the analysis included the conserved transcription factor binding sites retrieved from the UCSC Bioinformatics website, Ensemble Gene Annotations, ENCODE Gene Annotations, Cytogenetic Band Annotations and Segmental Duplication Annotations. We also compared the Malay genome with other personal genomes which include those of the Sequence Ontology project [31] and two Indian genomes [8], [14] in order to determine the similarity of the genome with publically available personal genomes.

### Dataset of Genes Involved in Drug Transport, Metabolism and Drug Targets

A comprehensive dataset of the human genes involved in drug transport and metabolism including drug targets were retrieved from DrugBank [32], which was used for creating a comprehensive pathway map of drug transport, metabolism and action. The datasets were eventually used as the template to map non-synonymous variations with potential functional consequences. This pathway map is available at OpenPGx in Systems Biology Markup Language (SBML) format [www.openpgx.org]. An independent dataset of genes having functional annotations in drug transport, metabolism and drug targets was retrieved from PharmGKB [33].

### Analysis of Genomic Variations Associated with Disease and Pharmacogenomics Traits

The clinically relevant pharmacogenomics variant datasets were derived from PharmGKB [33] . Additionally, we compared a comprehensive set of genetic markers associated with pharmacogenomics traits manually curated from literature and public databases against the variants called from the genome. The overlaps between the two sets were computed using custom scripts in Perl. The scripts are available for download at the OpenPGx website [www.openpgx.org]. Subsets of the variations have high strength of association and have been clinically recommended for testing.

In addition, disease associated traits compiled as part of the NHGRI GWAS catalog was also mapped to the variant dataset derived from the Malay genome. The mappings were performed using bespoke scripts. The risk magnitude of each of the mapped candidate variants were further compiled from a public resource SNPedia [34].

### Analysis of Potential Deleterious Non-Synonymous Single Nucleotide Variations (SNVs) in Genes Involved in Drug Transport, Metabolism or Drug Targets

All nonsynonymous variations of the Malay genome were mapped to the exons of the coding RefSeq genes and were further analyzed for their potential functional effects using two popular bioinformatics tools: Sorting Intolerant from Tolerant (SIFT) [35] and Polyphen-2 [36]. All coding non-synonymous single nucleotide variations (SNVs) were filtered from the annotations against the RefSeq gene loci using ANNOVAR. The annotation file was transformed into input format conforming to SIFT and PolyPhen-2 using custom script written in Perl. The annotations of SIFT and PolyPhen2 were compiled and a consensus annotation was derived for the functional effects. The genes with significant variations in the functional effects were further mapped to the DrugBank. This includes the list of genes involved in drug transport, metabolism and drug targets.

### Pathway Construction for Drug Pathways

A comprehensive map of drugs, their transporters, metabolizing enzymes and targets were compiled from DrugBank and transformed according to the Systems Biology Markup Language (SBML) format. This comprehensive dataset was compiled as part of the OpenPGx initiative. The dataset has information on a total of 6,707 drugs and therapeutic agents with details of 3,375 genes involved in their transport and metabolism including drug targets. This comprehensive pathway map was used as a template to analyse the drugs and genes, which could have potential functional effects due to the deleterious variations in the genome. Deleterious variants predicted by the consensus approach as described above were mapped to the drug pathways to understand how the variants could potentially contribute to altered transport, metabolism and targets of drugs.

### Human Genome Variation Society (HGVS) Nomenclature and Mapping

The genomic variants in the Malay genome mappped to genes in the DrugBank were transformed to HGVS nomenclature using a set of custom scripts in Perl based on the RefSeq database co-ordinates. The scripts used for the conversion are made available on the OpenPGx website.

### Data Visualization and Rendering

The genomic variations and information on the associated annotation have been made available for easy visualization using the Open Source Gbrowse [37] software and features a browse-able web based interface. The summary for the visualization of the genetic SBML data for pathways was done using cell designer [38] and the drug pathway was plotted using Sankey diagrams and Microsoft Excel plugins [http://ramblings.mcpher.com].

## Results

### DNA Sample and Quantitation

DNA was isolated from the blood collected following venipuncture under aseptic precautions after obtaining consent from the subject as per the guidelines laid down by the Institutional Ethics review committee. The library was prepared after DNA purification and fragmentation, followed by quantitation using 2100 (Agilent Technologies, California, USA) as detailed in the Materials and Methods section and Figure S2.

### Sequencing and Reference Alignments

A total of 2,872,434,354 paired-end reads of 100 nucleotides length were generated, out of which nearly 76% of the filtered reads mapped to the reference genome covering 70.60 fold effective depth. Alignment and comparison of variants with respect to the hg19 build of the human reference genome revealed approximately 3.5 million SNVs in the Malay genome. This included 1,545,544 homozygous variants and 1,998,216 heterozygous variations. We also identified a number of small insertions and deletions amounting to 238,287 in number.

### Primary Analysis of the SNVs

A newly developed computational pipeline was used for the analysis of the SNVs as detailed in the Materials and Methods section, which involved a primary analysis and gene/locus mapping followed by an in-depth analysis of the pharmacologically relevant variations (Figure 1). The initial component included the comparison of the SNVs in the Malay genome against other public databases of genomic variations. Three large datasets were used for this comparison, which includes the dbSNP, which comprising of 53,502,122 variations curated from multiple sources, datasets from the 1000 Genomes project and variant datasets from 11 personal genome sequences previously published.

**Figure 2:**
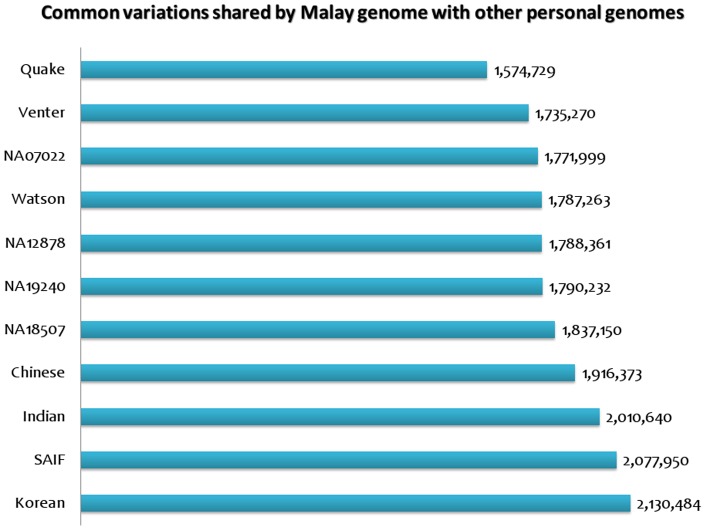
Comparative SNV analysis of other personal genomes with the Malaysian genome.

Our analysis of variants in the Malay Genome revealed that over 3 million of the variations in the Malay genome mapped to known variations within dbSNP while a total of 3,188,408 of 42,074,823 SNVs overlapped with the 1000 Genomes datasets. In summary, we identified 100,898 novel variations in the Malay genome through a sequential filtering approach, the summary of which is listed in Table 1. Further comparative analysis of the Malay genome with other personal genomes showed that it is more closely related to the Korean genome as compared to the other Caucasian genomes. The Indian genomes were the next closest relatives of the Malay genomes. Concise results depicting comparison is shown in Figure 2 and summarized in Table S1.

**Table 1:** Summary of SNVs found in the Malay genome and overlaps with dbSNP and 1000 Genome datasets.

Total SNVs	Homozygous SNVs	% of homozy cgous SNVs	Heterozygous SNVs	% of heterozygous SNVs	SNVs mapped to dbSNP (v135)	% of SNVs mapped to dbSNP (v135)	SNVs mapped to 1000 Genome dataset	% of SNVs mapped to 1000 Genome dataset	Novel SNVs	% of Novel SNVs
3,543,760	1,545,544	43.61%	1,998,216	56.38%	3,300,328	95.64%	3,188,408	92.40%	100,898	2.92%

The Malay genome was further compared with genome annotation datasets available in public domain. Analysis revealed 1,449,995 variations mapped to the genic regions, of which 19,896 mapped to the exonic regions while 1,267,891 mapped to the introns. A further analysis revealed that 4,309 and 23,675 variants mapped to the 5′ and 3′ un-translated regions respectively. As expected, majority of the variations fell in the intergenic and intronic regions of the genome. Of the total 1,449,995 genic variations; 10,191 variants were nonsense mutations while 9,142 of the variants which mapped to the exonic region of the protein coding genes were non-synonymous. A small but significant number of the insertions, deletions and substitutions gave rise to frame shift mutations in the genic region of the genome. The results of the mapping with respect to the genic loci are summarized in Table 2 and detailed in Table S2.

**Table 2:** Database mapping of SNVs found in the Malay individual.

SNVs Mapping	Number of Variants
Total SNVs*	3,543,760
Novel SNVs	100,898
Novel Indels	147,894
SNVs mapping to Exonic regions^$^	19,896
SNVs found in 3′ UTR^$^	23,675
SNVs found in 5′ UTR^$^	4,309
Synonymous Variants^$^	10,191
Nonsynonymous variants^$^	9,142
SNVs with StopGain^$^	87
SNVs with StopLoss^$^	42

$: Positioning of variations to genomic loci with respect to RefGene.

* : Including indels.

### Analysis of Clinically Relevant Pharmacogenomics Markers

A comprehensive analysis of the pharmacogenomics markers was also performed for 411 genetic markers and over 576 drugs from DrugBank. Analyses revealed 131 markers in the Malay Genome corresponding to 131 genes and 187 drugs. Our analysis revealed three variants associated with warfarin dose response and 1 variant associated with toxicity/adverse drug reactions of phenytoin. The variants, drugs type and strength of evidence for all the variants are summarized in Table S3.

Analysis using PharmGKB revealed genomic variations in the Malay genome which corresponded to 174 unique genes and 101 unique drugs. A large number of these traits were found to be associated with drug toxicity, while others belonged to drugs used in the treatment of diseases such as depression, hypertension, schizophrenia and other psychotic disorders. The compilation of genomic variations in the Malay genome and their potential associations with pharmacogenomics traits are summarized in Table S4.

### Analysis of Potential Deleterious Variations in Genes Involved in Drug Transport, Metabolism and Targets

SIFT analysis revealed 1,483 variants in 1,206 proteins that could have possible deleterious effects while the PolyPhen-2 analysis indicated 1,196 variants in 1,016 genes as possibly damaging. To improve the confidence of the potential damaging variations, we used a consensus of both tools, revealing 607 unique variants in 563 proteins. The results of this analysis are summarized in Table 3. To analyze the potential impact of the identified deleterious variations with respect to drug transport, metabolism and targeting, we used a consensus prediction employing SIFT and PolyPhen-2 against a well annotated dataset of genes involved in pharmacologic pathways derived from DrugBank. Our analysis revealed 4 drug transport genes, 2 drug metabolizing enzymes and 33 drug targets to be harboring deleterious non-synonymous variations, many of which are involved in the transport, metabolism or targeting of drugs used as hematological, psychiatric, oncological, analgesic, antiviral and anti-infective agents. A complete list of associated drugs is summarized in Table S5. A similar analysis was performed for an independent dataset of genes derived from PharmGKB and is summarized in Table S6.

**Table 3:** Predicted number of potentially damaging and deleterious variants as predicted by computational tools SIFT and Polyphen-2.

Prediction Tool	Class Predicted	Number of Unique Proteins	Number of Unique Variants
SIFT	Damaging	1206	1483
Polyphen2	Benign	4615	7347
Polyphen2	Possibly Damaging	566	618
Polyphen2	Probably Damaging	522	578
Common Between SIFT & Polyphen2	-	563	607

### Pathway Mapping of Genes Harboring Deleterious SNVs

**Figure 3:**
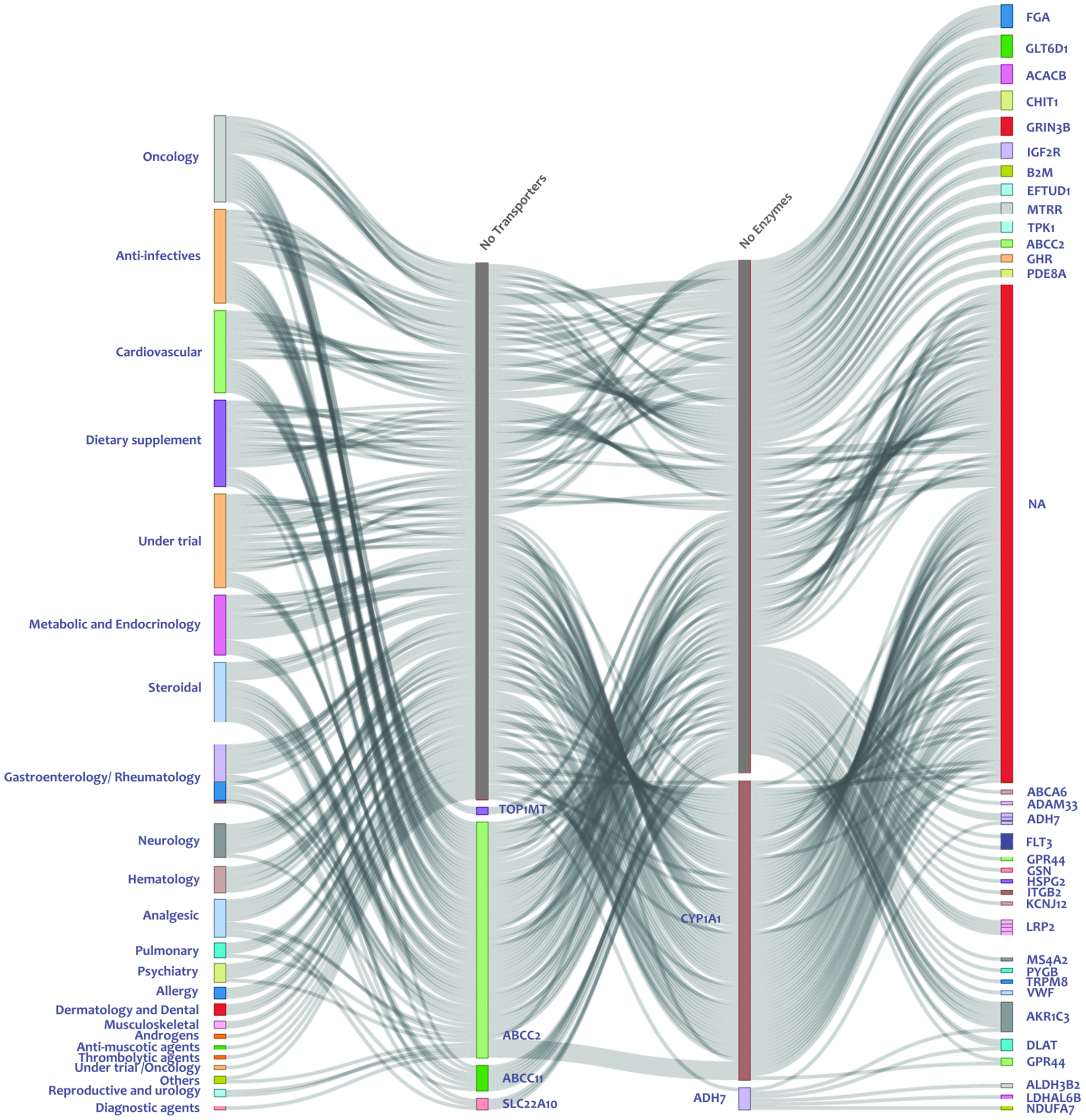
Flow diagram showing drug pathway for genes disrupted in the Malay genome. Disrupted transporters and targets are depicted in the second and fourth columns of the diagram while enzymes involved in pathways are shown in the third columns. First column shows the drugs which are affected due to disrupted genes in the Malay individual.

Potential deleterious variations in genes involved in drug transport, metabolism and targets were further mapped onto the pathway map curated from literature and public resources. This would provide a holistic approach to understand the pharmacokinetics-pharmacodynamics (PK-PD) pathways of drugs and offer clues towards the potential phenotypic impact of these variations with respect to off-target events, alternative pathways and potential side effects. Mapping variations to drug pathways are also relevant in the context as many drugs would have alternate pathways which could be exploited despite a deleterious effect on one gene. Additionally some genes could be important with respect to the transport or metabolism of multiple drugs. This complexity could be addressed through a visual representation of the deleterious genes vis-à-vis their function and context in the pathway. Our analysis shows deleterious variations in a number of drug-transport and metabolism genes encompassing a wide spectrum of drug classes ranging from anti-infective to anti-neoplastic agents. The largest number of drugs belonged to anti-infective and anti-neoplastic agents. We also find a significant number of genes related to pathways of drugs presently under trial. Such a map would potentially offer clinicians newer clues which could be explored, assayed and validated in detail and be used to prioritise drugs and effectively plan the therapy. For example nifedipine, a popular anti-anginal and anti-hypertensive and quinidine, an anti-arrhythmic had deleterious mutations in the transporter as well as the metabolizing enzymes in the individual which could be explored in detail with respect to the dose response or side effects. The data on individual drugs and genes are summarized in Figure 3.

### Analysis of Disease and Pharmacogenomics Markers in the Malay Individual for Potential Clinical Applications

**Figure 4:**
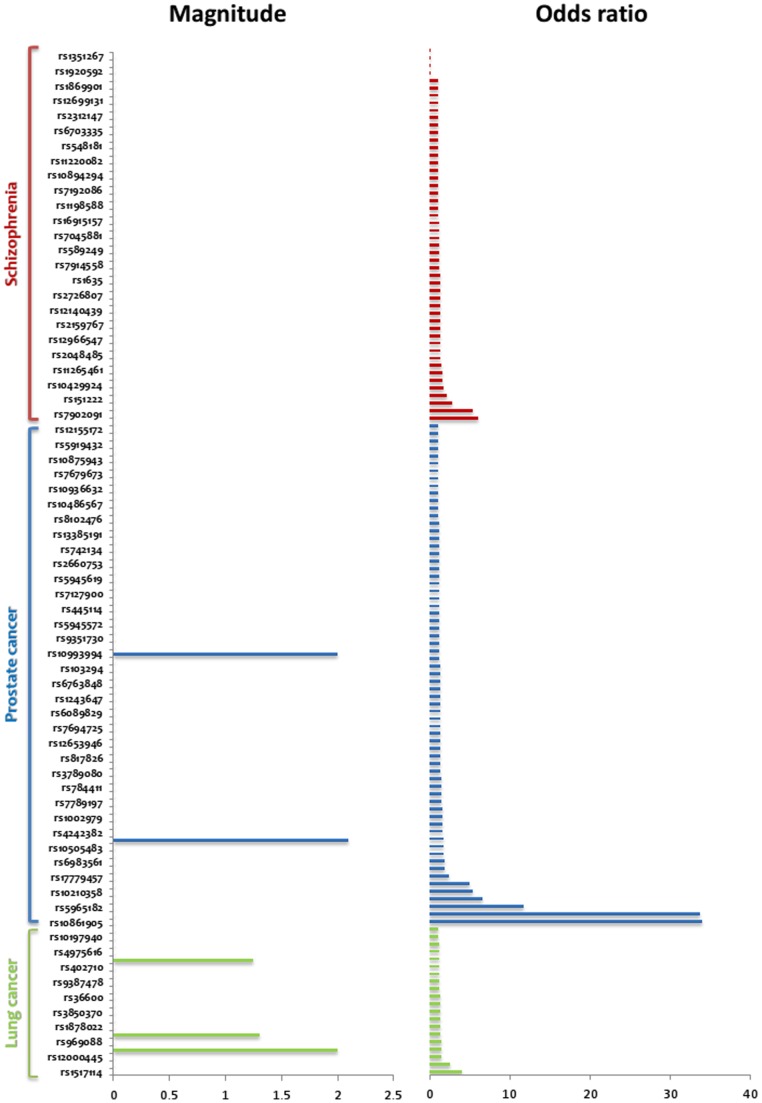
Disease risk plot for the Malay genome. The bars in blue show risk magnitude for alleles showing association with lung cancer while the bars in red show risk magnitude for prostate cancer associated alleles.

The subject had a history of prostate enlargement and a family history of schizophrenia and lung cancer and it was necessary to analyze disease markers which are associated with these diseases. Analysis identified presence of 67 markers out of 166 mentioned in the GWAS Catalog We additionally identified 50 markers for schizophrenia in the subject out of a total of 131 mentioned in the NHGRI GWAS Catalog. Similarly we analyzed the markers for lung cancer and identified 20 markers present in the genome of the subject out of 88 mentioned in NHGRI GWAS Catalog. Comparison of the risk magnitudes showed that 5 of the risk alleles for lung cancer had more than 1X risk magnitude while 4 of the prostate cancer risk alleles had more than 1.5X risk magnitude. No significant risk alleles with high magnitude for schizophrenia were observed in this individual. Figure 4 shows the risk alleles, magnitude from SNPedia and the odds ratios derived from the NHGRI GWAS catalog (Table S7). We further analyzed the pharmacogenomics information for the drugs recommended for these diseases in the genome of the subject. We found 13 pharmacogenomics markers in the genome of the subject associated with 4 drugs (risperidone, clozapine, olamzapine & haloperidol) recommended for schizophrenia and 7 markers associated with 4 drugs (docetaxel, paclitaxel, taxanes & thalidomide) prescribed for prostate neoplasm. We also found 2 markers for lung cancer associated with 1 drug (docetaxel) in the Malay genome. Therefore, the variable responses of these drugs used in this subject may be attributed by these pharmacogenomics markers.

## Discussions

While newer technologies claim to have made a giant leap forward with respect to the scale and cost of sequencing [1], there is still an immense scope for improvement in these facets of genome sequencing [39]. At the same time, there has been a spurt in the amount of data generated, which could have tremendous potential application in clinical settings but needs to be complemented by equally efficient computational systems which could mine this data in the near real-time. Not only does this field hold promise as an area under development, it also needs to be matched by improvements in the techniques of data mining and analysis. We hope that better focused workflows and methodologies would be available in the near future to conceptually mine and analyze data that would improve the usability and applicability of whole genome sequencing in clinical settings. Also, the availability of the workflows for improvement and modification would also be crucial in this setting.

In the present report, we describe a comprehensive computational pipeline for systematic data mining and analysis of personal genome data for variants with potential implications on PK-PD of drug pathways. We use an intuitive approach integrating both high quality datasets of genes and variant annotations curated from literature and computational methods to predict potential functionally relevant variations. In addition, special emphasis has been laid on analysing variants with established clinical relevance, including variations for which clinical testing has been recommended. We also integrate the analysis with PK-PD pathway information to potentially provide a holistic view of the implications of these variations.

This report is unique in many ways. Firstly, it reports a comprehensive computational pipeline for analysis of personal genome and applications in pharmacogenomics. Secondly, it provides data on genomic variations of a Malay individual which adds a subset of novel variations to the existing repertoire of genomic variations known in human populations. We hope this would be a starting point towards the sequencing of a larger number of individuals from the subcontinent region aimed at understanding the population frequencies of functionally relevant variations and genetic structure of the population, with far-reaching implications in healthcare planning and management. To accelerate the adoption of the methodology described and potential future modification and application in clinical settings, we have made available the resources in public domain through the OpenPGx consortium [www.openpgx.org]. We hope this would add to the methodology toolkit for data mining and analysis of personal genomes and accelerate the adoption and application of whole genome sequencing in clinical settings.

The personal genome datasets and information generated could help the Malay individual and his physician to strategize his health management plan as the genetic variation in enzymes of drug metabolism and transport are known. This allows optimization of drug use and to avoid inadequate therapeutic responses owing to the inability to absorb a drug, the inability to activate a pro-drug, or excessive metabolism and/or excretion of an active drug. Similarly, adverse reactions to a drug may be avoided as the genome encodes defective enzymes of drug metabolism, resulting in an abnormally high exposure to the drug despite normal dosing is uncovered. We analyzed the disease markers in the genome of the individual to identify probable risk factors to some common diseases observed in the familial history of the Malay genome. Also, based on these disease markers and familial history of the Malay genome we identified potential pharmacogenomics markers associated with drugs used for treatment of lung and prostate cancer.

This study is a proof of concept whereby success of the use of second generation sequencing technologies and systematic evaluation and analysis of personal genomes using the computational pipeline developed would help the realization of personalized medicine as widespread use of complete, genome-wide information on an individual basis can be applied into clinical practice. Disease traits and associated pharmacogenomics information for schizophrenia, lung cancer and prostate cancer identified here would help the Malay individual change his lifestyle and carry out preventive actions for a healthier life.

## Supporting Information

Figure S1 **Karyotyping result for the DNA genome sequenced.**
(TIF)

Figure S2 **Results on the quality controls of the DNA prepared for sequencing.**
(DOCX)

Data S1 **Parameters for BWA and SAMtools used to call quality SNVs from the Malaysian genome.**
(DOCX)

Table S1 **SNV comparison of the Malay genome with other published personal genomes.**
(DOCX)

Table S2 **Database mapping of SNVs found in the Malay genome.**
(DOCX)

Table S3 **List of pharmacogenomics markers found in the Malay genome for which clinical testing is recommended.**
(DOCX)

Table S4 **List of pharmacogenomics markers positive in the Malay genome.**
(DOCX)

Table S5 **List of drug pathway genes harboring deleterious mutations and the drugs affected.**
(DOCX)

Table S6 **List of drugs (PharmGKB) implicated with the genetic variation of the Malay individual.**
(DOCX)

Table S7 **Risk alleles and corresponding risk magnitude and odds ratio.**
(DOCX)
