# Supplementary figures and images for: Systematic Pharmacogenomics Analysis of a Malay Whole Genome: Proof of Concept for Personalized Medicine
Source: PLoS One. 2013 Aug 23;8(8):e71554. doi: 10.1371/journal.pone.0071554 (PMC3751891; doi:10.1371/journal.pone.0071554)

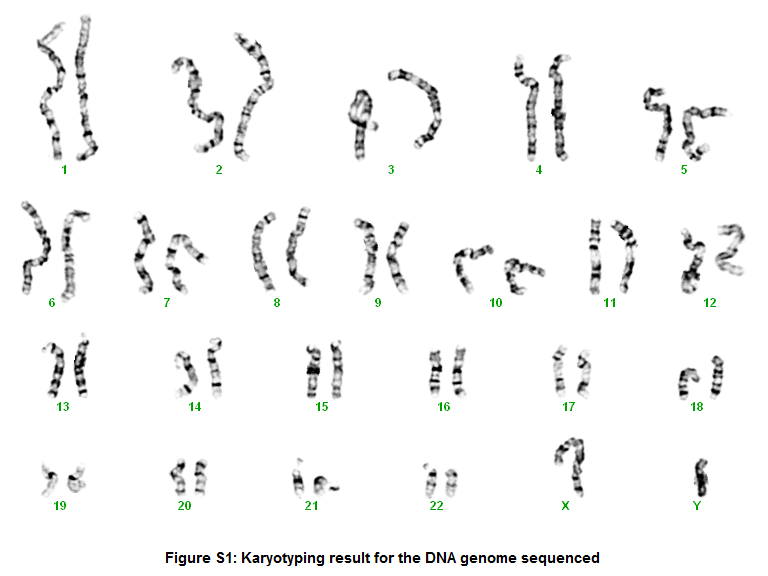

Supplement: Figure S1 — Karyotyping result for the DNA genome sequenced. (TIF) [file pone.0071554.s001.tif]
