# Supplementary material for: Systematic Pharmacogenomics Analysis of a Malay Whole Genome: Proof of Concept for Personalized Medicine
Source: PLoS One. 2013 Aug 23;8(8):e71554. doi: 10.1371/journal.pone.0071554 (PMC3751891; doi:10.1371/journal.pone.0071554)
Supplement: Figure S2 — Results on the quality controls of the DNA prepared for sequencing. (DOCX) [file pone.0071554.s002.docx]

**Figure 2S: Results on the quality controls of the DNA prepared for sequencing**

Supplementary Figure: Gel electrophoresis of genomic DNA used in sequencing


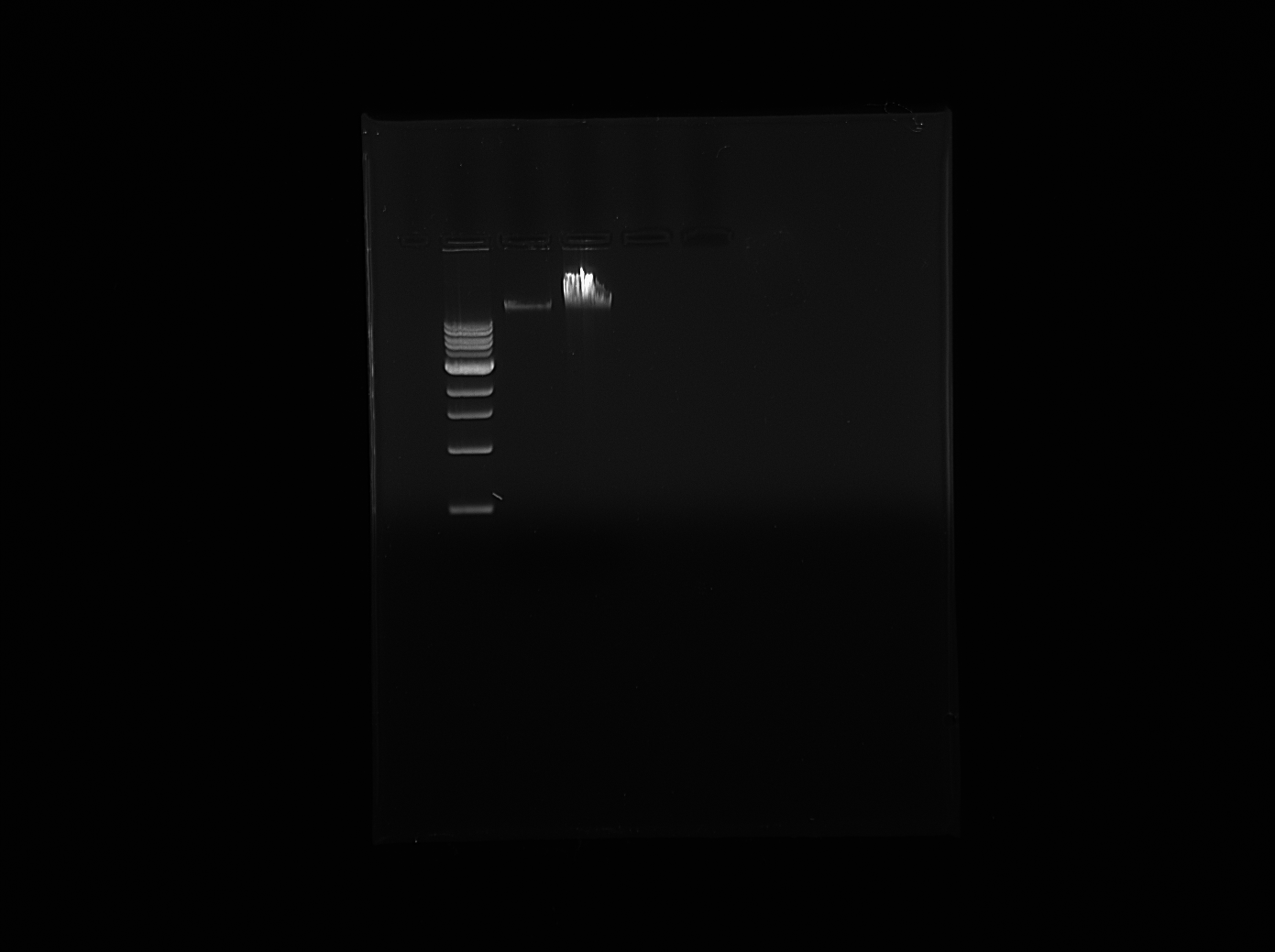


| Lane | Sample |
| --- | --- |
| 1 | 1 kb ladder |
| 2 | Human gDNA |

Supplementary Figure: Gel electrophoresis of DNA library used in sequencing (before 2^nd^ gel excision step).


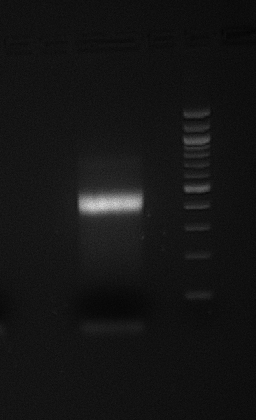


| Lane | Sample |
| --- | --- |
| 1 | DNA library H3 |
| 2 | 100bp ladder |

Bioanalyzer result:

**
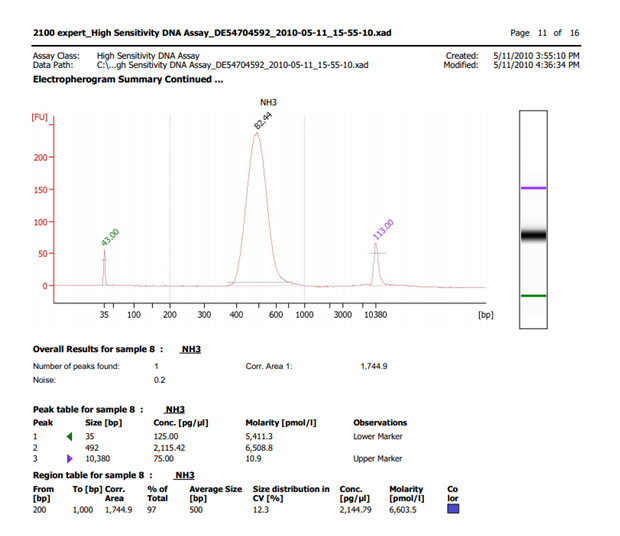
**

Qubit concentration of DNA library: 7.527 ng/ul
