# Supplementary material for: Systematic Pharmacogenomics Analysis of a Malay Whole Genome: Proof of Concept for Personalized Medicine
Source: PLoS One. 2013 Aug 23;8(8):e71554. doi: 10.1371/journal.pone.0071554 (PMC3751891; doi:10.1371/journal.pone.0071554)
Supplement: Data S1 — Parameters for BWA and SAMtools used to call quality SNVs from the Malaysian genome. (DOCX) [file pone.0071554.s003.docx]

$ samtools mpileup -ugf hg19.fasta sorted.bam | bcftools view -bvcg - > var.raw.bcf

$ bcftools view var.raw.bcf | vcfutils.pl varFilter -D 150 > var.flt.vcf

$ awk '($3=="*"&&$6>=50)||($3!="*"&&$6>=20)' var.flt.vcf > var.flt.final.txt
