# Supplementary material for: Systematic Pharmacogenomics Analysis of a Malay Whole Genome: Proof of Concept for Personalized Medicine
Source: PLoS One. 2013 Aug 23;8(8):e71554. doi: 10.1371/journal.pone.0071554 (PMC3751891; doi:10.1371/journal.pone.0071554)
Supplement: Table S1 — SNV comparison of the Malay genome with other published personal genomes. (DOCX) [file pone.0071554.s004.docx]

Table S1: SNV comparison of Malay genome with other published personal genomes

| Personal genome | Common SNVs with Malaysian genome |
| --- | --- |
| Korean | 2130484 |
| SAIF | 2077950 |
| Indian | 2010640 |
| Chinese | 1916373 |
| NA18507 | 1837150 |
| NA19240 | 1790232 |
| NA12878 | 1788361 |
| Watson | 1787263 |
| NA07022 | 1771999 |
| Venter | 1735270 |
| Quake | 1574729 |
