# Supplementary material for: Systematic Pharmacogenomics Analysis of a Malay Whole Genome: Proof of Concept for Personalized Medicine
Source: PLoS One. 2013 Aug 23;8(8):e71554. doi: 10.1371/journal.pone.0071554 (PMC3751891; doi:10.1371/journal.pone.0071554)
Supplement: Table S2 — Database mapping of SNVs found in the Malay genome. (DOCX) [file pone.0071554.s005.docx]

**Table S2: Database mapping of SNVs found in the Malay individual.**

| **SNVs Mapping** | **Number of Variants** |
| --- | --- |
| **Total SNVs^*^** | **3,543,760** |
| **SNVs mapping to dbSNP (v135)^1^** | **3,300,328** |
| **Indel mapping to dbSNP (v135)^1^** | **225,927** |
| **Novel SNVs** | **100,898** |
| **Novel Indels** | **147,894** |
| **SNVs mapping to 1000Genome ALLSites data (Feb 2012)^2^** | **3,224,105** |
| **SNVs mapping to Intergenic regions^3^** | **2,293,388** |
| **SNVs mapping to Intronic regions^3^** | **1,267,891** |
| **SNVs mapping to Exonic regions^3^** | **19,896** |
| **SNVs found in 3' UTR^3^** | **23,675** |
| **SNVs found in 5' UTR^3^** | **4,309** |
| **SNVs mapped Downstream to RefGene^3^** | **23,069** |
| **SNVs mapped Upstream to RefGene^3^** | **21,413** |
| **Synonymous Variants^3^** | **10,191** |
| **Nonsynonymous variants^3^** | **9,142** |
| **SNVs with StopGain^3^** | **87** |
| **SNVs with StopLoss^3^** | **42** |
| **Frameshift Deletions^3^** | **70** |
| **NonFrameshift Deletions^3^** | **109** |
| **Frameshift Insertions^3^** | **100** |
| **NonFrameshift Insertion^3^** | **91** |
| **Frameshift Substitution^3^** | **2** |
| **NonFrameshift Substitution^3^** | **8** |
| **SNVs mapped to Database of Genomic Variants^4^** | **1,337,819** |
| **SNVs mapped to GWASCatalog^5^** | **2,849** |
| **SNVs mapped to Transcription Factor Binding Sites^6^** | **43,300** |

**Where:**

**1: SNV mapping to dbSNP (v135)**

**2: SNV mapping to1000Genome data (release Feb 2012)**

**3: Positioning of variations to genomic loci with respect to RefGene**

**4: SNV mapping to Database of Genomic Variants**

**5: SNV mapping to GWAS Catalog**

**6: SNV mapping to Transcription factor binding sites**

***: Including indels**
