# Supplementary material for: Systematic Pharmacogenomics Analysis of a Malay Whole Genome: Proof of Concept for Personalized Medicine
Source: PLoS One. 2013 Aug 23;8(8):e71554. doi: 10.1371/journal.pone.0071554 (PMC3751891; doi:10.1371/journal.pone.0071554)
Supplement: Table S3 — List of pharmacogenomics markers found in the Malay genome for which clinical testing is recommended. (DOCX) [file pone.0071554.s006.docx]

**Table S3: List of Pharmacogenomics markers found in the Malay genome for which clinical testing is recommended.**

| **dbSNP Variant** | **Gene** | **PGx Type** | **Strength** | **Drugs involved** | **Drug Class** | **Genetic Tests Available** |
| --- | --- | --- | --- | --- | --- | --- |
| **rs2238472** | ABCC6 | T/ADR | 3 | Docetaxel,Thalidomide | Oncology | - |
| **rs1045642** | ABCB1 | E | 3 | Anthracyclines and related substances,Taxanes |  | - |
| **rs1883322** | PPARD | E | 3 | Docetaxel,Thalidomide |  |  |
| **rs10821936** | ARID5B | O | 3 | Methotrexate |  | - |
| **rs3734254** | PPARD | E | 3 | Docetaxel,Thalidomide |  | - |
| **rs396991** | FCGR3A | E | 2 | Rituximab |  | - |
| **rs13181** | ERCC2,KLC3 | E | 3 | Cisplatin |  | - |
| **rs8133052** | CBR3 | E | 2 | Doxorubicin |  | - |
| **rs2016520** | PPARD | E | 3 | Docetaxel,Thalidomide |  | - |
| **rs2070744** | NOS3 | E | 2 | Cyclophosphamide,Doxorubicin,Fluorouracil,Methotrexate |  | - |
| **rs2075252** | LRP2 | T/ADR | 3 | Cisplatin |  |  |
| **rs1695** | GSTP1 | E | 3 | Platinum compounds |  | - |
| **rs1402467** | SULT1C4 | E | 3 | Docetaxel,Thalidomide |  | - |
| **rs2032582** | ABCB1 | E | 3 | Paclitaxel |  | - |
| **rs4646** | CYP19A1 | E | 3 | Letrozole |  | - |
| **rs25487** | XRCC1 | T/ADR | 2 | Cisplatin,Cyclophosphamide |  | - |
| **rs3957357** | GSTA1 | E | 2 | Cisplatin,Cyclophosphamide |  | - |
| **rs714368** | SLC22A16 | D,T/ADR | 3 | Doxorubicin,Doxorubicinol |  | - |
| **rs9024** | CBR1,SETD4 | D | 3 | Doxorubicin |  | - |
| **rs1056836** | CYP1B1 | E | 2 | Docetaxel,Paclitaxel,Taxanes |  | - |
| **rs2278293** | IMPDH1 | T/ADR | 2 | Mycophenolate mofetil |  | - |
| **rs870995** | PIK3CA | D | 3 | Docetaxel |  | - |
| **rs3212986** | CD3EAP,ERCC1,PPP1R13L | T/ADR | 3 | Cisplatin,Cyclophosphamide |  | - |
| **rs1799983** | NOS3 | E | 2 | Cyclophosphamide,Doxorubicin,Fluorouracil,Methotrexate |  | - |
| **rs11045879** | SLCO1B1 | T/ADR | 2 | Methotrexate |  | - |
| **rs4888024** |  | T/ADR | 2 | Methotrexate |  | - |
| **rs10836235** | CAT | T/ADR | 3 | Anthracyclines and related substances |  | - |
| **rs1113129** | CYP2C8 | T/ADR | 3 | Paclitaxel |  | - |
| **rs776746** | CYP3A,CYP3A5 | T/ADR | 3 | Paclitaxel |  | - |
| **rs9561778** | ABCC4 | T/ADR | 2 | Cyclophosphamide |  | - |
| **rs11615** | ERCC1 | T/ADR | 3 | Cisplatin,Cyclophosphamide |  | - |
| **rs2784917** | SLIT1 | T/ADR | 3 | Etoposide |  | - |
| **rs4149081** | SLCO1B1 | T/ADR | 2 | Methotrexate |  | - |
| **rs1382368** | XRCC4 | D | 3 | Docetaxel |  | - |
| **rs9344** | CCND1 | E | 3 | Cetuximab |  | - |
| **rs316019** | SLC22A2 | T/ADR | 3 | Cisplatin |  | - |
| **rs1799793** | ERCC2 | T/ADR | 3 | Cisplatin,Cyclophosphamide |  | - |
| **rs1799931** | -T2 | T/ADR | 3 | Docetaxel,Thalidomide |  | - |
| **rs2228001** | TMEM43,XPC | T/ADR | 3 | Cisplatin |  | - |
| **rs20572** | CBR1,SETD4 | D | 3 | Doxorubicin |  | - |
| **rs1042522** | TP53,WDR79,WRAP53 | E | 3 | Fluorouracil |  | - |
| **rs1800909** | GGH | E | 3 | Methotrexate | Rheumatology | - |
| **rs1801131** | MTHFR | E | 3 | Methotrexate |  | - |
| **rs5760410** | ADORA2A,CYTSA | T/ADR | 2 | Methotrexate |  | - |
| **rs2297480** | FDPS,PKLR | E | 3 | Bisphospho-tes |  | - |
| **rs854548** | PON1,PPP1R9A | E | 3 | Tumor necrosis factor alpha (TNF-alpha) inhibitors |  | - |
| **rs868856** | MOBKL2B | E | 3 | Tumor necrosis factor alpha (TNF-alpha) inhibitors |  | - |
| **rs928655** | GBP6 | E | 3 | Tumor necrosis factor alpha (TNF-alpha) inhibitors |  | - |
| **rs3849942** | C9orf72 | E | 3 | Tumor necrosis factor alpha (TNF-alpha) inhibitors |  | - |
| **rs4846051** | MTHFR | T/ADR | 3 | Methotrexate |  | - |
| **rs2853539** | C18orf56,TYMS | E | 2 | Methotrexate |  | - |
| **rs1051266** | SLC19A1 | E | 2 | Methotrexate |  | - |
| **rs7046653** | MOBKL2B | E | 3 | Tumor necrosis factor alpha (TNF-alpha) inhibitors |  | - |
| **rs1934951** | CYP2C8 | T/ADR | 3 | Pamidro-te,Zoledro-te |  | - |
| **rs2236624** | ADORA2A | T/ADR | 2 | Methotrexate |  | - |
| **rs3761422** | ADORA2A | T/ADR | 2 | Methotrexate |  | - |
| **rs16944** | IL1B | E | 2 | Bisphospho-tes,Clodro-te,Etidronic acid,Risedro-te,Tiludro-te |  | - |
| **rs2298383** | ADORA2A | T/ADR | 2 | Methotrexate |  | - |
| **rs2267076** | ADORA2A | T/ADR | 2 | Methotrexate |  | - |
| **rs2814707** | MOBKL2B | E | 3 | Tumor necrosis factor alpha (TNF-alpha) inhibitors |  | - |
| **rs742105** | DTNBP1 | E | 3 | Clozapine | Psychiatry | - |
| **rs3813929** | HTR2C | T/ADR | 2 | Olanzapine |  | - |
| **rs1360780** | FKBP5 | E | 2 | Antidepressants |  | - |
| **rs4731426** | LEP | T/ADR | 2 | Olanzapine |  | - |
| **rs7997012** | HTR2A | T/ADR | 3 | Olanzapine |  | - |
| **rs6295** | HTR1A | E | 3 | Antidepressants |  | - |
| **rs334558** | GSK3B | E | 2 | Citalopram,Fluoxetine |  | - |
| **rs909706** | DTNBP1 | E | 3 | Clozapine,Haloperidol |  | - |
| **rs1954787** | GRIK4 | E | 1 | Citalopram |  | - |
| **rs2661319** | RGS4 | E | 2 | Risperidone |  | - |
| **rs1799732** | DRD2 | E | 3 | Antipsychotics,Clozapine,Olanzapine,Risperidone |  | - |
| **rs2235015** | ABCB1 | E | 3 | Antidepressants,O antidepressants |  | - |
| **rs2284017** | CACNG2 | E | 2 | Lithium |  | - |
| **rs1128503** | ABCB1 | E | 3 | Risperidone |  | - |
| **rs724226** | GRM3 | E | 2 | Risperidone |  | - |
| **rs3813928** | HTR2C | E | 3 | Risperidone |  | - |
| **rs10042486** | HTR1A | E | 2 | Fluvoxamine,Mil-cipran,Paroxetine |  | - |
| **rs2494732** | AKT1 | E | 2 | Risperidone |  | - |
| **rs1414334** | HTR2C | T/ADR | 2 | Antipsychotics,Clozapine,Risperidone |  | - |
| **rs167771** | DRD3 | T/ADR | 3 | Risperidone |  | - |
| **rs518147** | HTR2C | T/ADR | 2 | Olanzapine |  | - |
| **rs762551** | CYP1A2 | O | 3 | Olanzapine |  | - |
| **rs3812718** | SCN1A | D | 2 | Carbamazepine |  | - |
| **rs2227631** | SERPINE1 | E | 2 | Antidepressants,Citalopram,Fluoxetine |  | - |
| **rs2076369** | PICK1 | T/ADR | 2 | Methamphetamine |  | - |
| **rs3800373** | FKBP5 | E | 2 | Antidepressants |  | - |
| **rs2842030** | RGS4 | E | 3 | Risperidone |  | - |
| **rs7799039** | LEP | T/ADR | 3 | Risperidone |  | - |
| **rs2032583** | ABCB1 | E | 2 | Antidepressants,O antidepressants |  | - |
| **rs2108622** | CYP4F2 | D | 2 | Acenocoumarol,Warfarin | Cardiovascular | - |
| **rs12050217** | BDKRB1 | E | 2 | Perindopril |  | - |
| **rs2016848** | MME | T/ADR | 2 | Ace Inhibitors, Plain |  | - |
| **rs3745009** | SLC14A2 | E | 2 | Nifedipine |  | - |
| **rs4799915** | BRUNOL4,CELF4 | T/ADR | 3 | Iloperidone |  | - |
| **rs10918594** | NOS1AP | T/ADR | 3 | Verapamil |  | - |
| **rs1042714** | ADRB2 | E | 3 | Carvedilol |  | - |
| **rs1367117** | APOB | E | 3 | Irbesartan |  | - |
| **rs9934438** | PRSS53,VKORC1 | D | 2 | Acenocoumarol |  | - |
| **rs4933824** | NRG3 | T/ADR | 3 | Iloperidone |  | - |
| **rs7142881** | NUBPL | T/ADR | 3 | Iloperidone |  | - |
| **rs8012552** | BDKRB2 | T/ADR | 2 | Ace Inhibitors, Plain |  | - |
| **rs4673** | CYBA | T/ADR | 2 | Doxorubicin |  | - |
| **rs622342** | SLC22A1 | E | 2 | Metformin | Metabolic and Endocrinology | - |
| **rs2368564** | ETNK2,REN | T/ADR | 2 | Muraglitazar |  | - |
| **rs5443** | GNB3 | E | 3 | Atorvastatin,Fluvastatin,Hmg CoA reductase inhibitors,Lovastatin,Pravastatin,Rosuvastatin,Simvastatin |  | - |
| **rs4149015** | SLCO1B1 | E | 3 | Pravastatin |  | - |
| **rs5370** | EDN1 | T/ADR | 2 | Muraglitazar |  | - |
| **rs3846662** | HMGCR | O | 3 | Simvastatin |  | - |
| **rs2306283** | SLCO1B1 |  | 3 | Pravastatin |  | - |
| **rs2231142** | ABCG2 | E | 2 | Rosuvastatin |  | - |
| **rs9923231** | PRSS53,VKORC1 | D | 1 | Warfarin* | Hematology | TrimGen Corporation eQ-PCR LC Warfarin Genotyping Kit |
| **rs8050894** | PRSS53,VKORC1 | D | 2 | Warfarin* |  | TrimGen Corporation eQ-PCR LC Warfarin Genotyping Kit |
| **rs10871454** | STX4 | D | 2 | Warfarin |  | - |
| **rs2292566** | EPHX1 | D | 3 | Warfarin |  | - |
| **rs9934438** | PRSS53,VKORC1 | D | 2 | Warfarin* |  | TrimGen Corporation eQ-PCR LC Warfarin Genotyping Kit |
| **rs4086116** | CYP2C9 | D | 3 | Acenocoumarol |  | - |
| **rs2781659** | ARG1 | E | 2 | Budesonide,Fluticasone propionate,Nedocromil,Salbutamol | Pulmonary | - |
| **rs1042713** | ADRB2 |  | 3 | Salbutamol |  | - |
| **rs2115819** | ALOX5 | E | 3 | Montelukast |  | - |
| **rs2267715** | CRHR2 | E | 2 | Salbutamol,Selective beta-2-adrenoreceptor agonists |  | - |
| **rs255100** | CRHR2 | E | 2 | Salbutamol,Selective beta-2-adrenoreceptor agonists |  | - |
| **rs2660845** | LTA4H | E | 3 | Montelukast |  | - |
| **rs5128** | APOA1,APOA4,APOC3 | T/ADR | 2 | Ritonavir | Antivirals/Antiinfectives | - |
| **rs3775291** | TLR3 | E | 3 | Measles vaccines |  | - |
| **rs2740574** | CYP3A,CYP3A4 | O | 3 | Indinavir |  | - |
| **rs1044396** | CHR-4 | O | 2 | Nicotine | Neuorology | - |
| **rs2236196** | CHR-4 | O | 2 | Nicotine |  | - |
| **rs1051740** | EPHX1 | T/ADR | 2 | Phenytoin* |  | Roche AmpliChip CYP450 Test and Affymetrix GeneChip System 3000Dx, Infiniti CYP450 2C19, HLA-B*1502 Carbamazepine Sensitivity |
| **rs10115383** |  | O | 2 | Nicotine |  | - |
| **rs35592** | ABCC1 | E | 2 | Methotrexate | Dermatology and Dental | - |
| **rs246240** | ABCC1 | T/ADR | 2 | Methotrexate |  | - |
| **rs2070995** | KCNJ6 | E | 2 | Analgesics | Analgesics | - |

**Legend**

*** indicates a drug which has a Pharmacogenomics Test listed with the FDA. The different PGx types mentioned in the table are T=Toxicity, E=Efficacy, D=Dosage, ADR=Adverse Drug Reaction, O=Others.**
