# Supplementary material for: Systematic Pharmacogenomics Analysis of a Malay Whole Genome: Proof of Concept for Personalized Medicine
Source: PLoS One. 2013 Aug 23;8(8):e71554. doi: 10.1371/journal.pone.0071554 (PMC3751891; doi:10.1371/journal.pone.0071554)
Supplement: Table S4 — List of pharmacogenomics markers positive in the Malay genome. (DOCX) [file pone.0071554.s007.docx]

**Table S4: List of Pharmacogenomics markers positive in the Malay individual**

| RSID | Genes | Feature | Drugs | Drug Classes | Diseases |
| --- | --- | --- | --- | --- | --- |
| rs9934438 | VKORC1 | Intron | acenocoumarol |  | Arteriosclerosis; Heart Diseases; Hemorrhage; Intracranial Hemorrhages; Myocardial Infarction; Peripheral Vascular Diseases; Pulmonary Embolism; Stroke; Thromboembolism; venous thromboembolism; Venous Thrombosis |
| rs928655 | GBP6 |  | adalimumab; etanercept; infliximab |  | Arthritis, Rheumatoid |
| rs854548 | PPP1R9A, PON1 |  | adalimumab; etanercept; infliximab |  | Arthritis, Rheumatoid |
| rs2814707 | MOBKL2B |  | adalimumab; etanercept; infliximab |  | Arthritis, Rheumatoid |
| rs7046653 | MOBKL2B |  | adalimumab; etanercept; infliximab |  | Arthritis, Rheumatoid |
| rs868856 | MOBKL2B |  | adalimumab; etanercept; infliximab |  | Arthritis, Rheumatoid |
| rs2297480 | PKLR, FDPS |  | alendronate; ibandronate; pamidronate; risedronate; zoledronate |  | Osteoporosis; Osteoporosis, Postmenopausal |
| rs2235040 | ABCB1 | Intron | amitriptyline; citalopram; paroxetine; venlafaxine |  | Depression |
| rs12720067 | ABCB1 |  | amitriptyline; citalopram; paroxetine; venlafaxine |  | Depression |
| rs2235015 | ABCB1 | Intron | amitriptyline; citalopram; paroxetine; venlafaxine |  | Depression |
| rs2032583 | ABCB1 |  | amitriptyline; citalopram; paroxetine; venlafaxine |  | Depression |
| rs10248420 | ABCB1 |  | amitriptyline; citalopram; paroxetine; venlafaxine |  | Depression |
| rs4148739 | ABCB1 |  | amitriptyline; citalopram; paroxetine; venlafaxine |  | Depression |
| rs11983225 | ABCB1 |  | amitriptyline; citalopram; paroxetine; venlafaxine |  | Depression |
| rs7787082 | ABCB1 |  | amitriptyline; citalopram; paroxetine; venlafaxine |  | Depression |
| rs10280101 | ABCB1 |  | amitriptyline; citalopram; paroxetine; venlafaxine |  | Depression |
| rs2246709 | CYP3A4, CYP3A | Intron, NA | amlodipine |  | Hypertension |
| rs1367117 | APOB | Exon/NonSyn | atenolol; irbesartan |  | Hypertension |
| rs1051375 | CACNA1C | Exon/Syn | atenolol; verapamil | BETA BLOCKING AGENTS; CALCIUM CHANNEL BLOCKERS | Coronary Artery Disease; Death; Death, Sudden, Cardiac; Hypertension; Myocardial Infarction; Stroke |
| rs1042718 | ADRB2 | Exon/Syn | atenolol; verapamil |  | Coronary Artery Disease; Death; Hypertension; Myocardial Infarction; Stroke |
| rs4961 | ADD1 | Exon/NonSyn | benazepril |  | Hypertension |
| rs11545077 | GGH | Exon/NonSyn | bevacizumab; pemetrexed |  | Carcinoma, Non-Small-Cell Lung |
| rs1051298 | SLC19A1, COL18A1 |  | bevacizumab; pemetrexed |  | Carcinoma, Non-Small-Cell Lung |
| rs1050565 | BLMH |  | bleomycin |  | Testicular Neoplasms |
| rs2781659 | ARG1 |  | budesonide; fluticasone propionate; nedocromil; salbutamol |  | Asthma |
| rs28364072 | FCER2 | Intron | budesonide; nedocromil |  | Asthma |
| rs5030062 | KNG1 | Intron | candesartan; hydrochlorothiazide | ANTIHYPERTENSIVES |  |
| rs698078 | KNG1 | Intron | candesartan; hydrochlorothiazide | ANTIHYPERTENSIVES |  |
| rs699947 | VEGFA |  | capecitabine; oxaliplatin |  | Colorectal Neoplasms |
| rs2010963 | VEGFA |  | capecitabine; oxaliplatin |  | Colorectal Neoplasms |
| rs833061 | VEGFA |  | capecitabine; oxaliplatin |  | Colorectal Neoplasms |
| rs2395402 | LEMD2 |  | carbamazepine |  | Drug Hypersensitivity |
| rs3812718 | SCN1A |  | carbamazepine; phenytoin |  |  |
| rs13181 | ERCC2, KLC3 |  | cisplatin |  | Osteosarcoma |
| rs2075252 | LRP2 |  | cisplatin |  | nephrotoxicity; Ototoxicity |
| rs25487 | XRCC1 |  | cisplatin; cyclophosphamide |  | Neutropenia |
| rs3957357 | GSTAP1, GSTA1 |  | cisplatin; cyclophosphamide |  | Ovarian Neoplasms |
| rs1695 | GSTP1 | Exon/NonSyn | cisplatin; cyclophosphamide |  | Ovarian Neoplasms |
| rs1799793 | ERCC2 |  | cisplatin; cyclophosphamide |  | Neutropenia |
| rs351855 | FGFR4 |  | cisplatin; cyclophosphamide; fluorouracil; methotrexate; tamoxifen |  | Breast Neoplasms |
| rs1954787 | GRIK4 |  | citalopram |  | Depression |
| rs6808874 | GSK3B |  | citalopram; fluoxetine |  | Depression; Depressive Disorder, Major |
| rs2227631 | SERPINE1 |  | citalopram; fluoxetine |  | Depression; Depressive Disorder, Major |
| rs334558 | GSK3B |  | citalopram; fluoxetine |  | Depression; Depressive Disorder, Major; Parkinsonian Disorders |
| rs2319398 | GSK3B |  | citalopram; fluoxetine |  | Depression; Depressive Disorder, Major |
| rs13321783 | GSK3B |  | citalopram; fluoxetine |  | Depression; Depressive Disorder, Major |
| rs16944 | IL1B |  | clodronate; etidronic acid; risedronate; tiludronate |  | Osteitis Deformans |
| rs1041983 | NAT2 |  | clonazepam |  |  |
| rs1208 | NAT2 | Exon/NonSyn | clonazepam |  |  |
| rs2046934 | MED12L, P2RY12 |  | clopidogrel; ticlopidine |  |  |
| rs742105 | DTNBP1 |  | clozapine |  | Schizophrenia |
| rs3780412 | SLC1A1 |  | clozapine; olanzapine; risperidone |  | Obsessive-Compulsive Disorder; Schizophrenia |
| rs3780413 | SLC1A1 |  | clozapine; olanzapine; risperidone |  | Obsessive-Compulsive Disorder; Schizophrenia |
| rs4606 | RGS2 |  | clozapine; olanzapine; risperidone |  | Parkinson Disease; Psychotic Disorders |
| rs9561778 | ABCC4 |  | cyclophosphamide |  | Breast Neoplasms; Diarrhea; Leukopenia; Neutropenia |
| rs1799983 | NOS3 | Exon/NonSyn | cyclophosphamide; doxorubicin; fluorouracil; methotrexate |  |  |
| rs1128503 | ABCB1 | Exon/Syn | cyclosporine; digoxin; verapamil; vinblastine |  |  |
| rs1042597 | UGT1A8 | Exon/NonSyn | cyclosporine; mycophenolate mofetil; mycophenolic acid |  | Organ Transplantation |
| rs7311358 | SLCO1B3 | Exon/NonSyn | cyclosporine; mycophenolate mofetil; sirolimus; tacrolimus |  | Organ Transplantation |
| rs1800734 | EPM2AIP1, MLH1 |  | dacarbazine; procarbazine |  | therapy-related acute myeloid leukemia (t-ML) |
| rs3750518 | MAN1B1 |  | daunorubicin |  | Drug Toxicity |
| rs6603859 | C1orf144 |  | daunorubicin |  | Drug Toxicity |
| rs2296840 | PSMB4 |  | desipramine; fluoxetine |  | Depression |
| rs3800373 | FKBP5 |  | desipramine; fluoxetine; mirtazapine; venlafaxine |  | Depression |
| rs1360780 | FKBP5 |  | desipramine; fluoxetine; mirtazapine; venlafaxine |  | Depression |
| rs2236930 | IL1R2 |  | docetaxel |  |  |
| rs870995 | PIK3CA |  | docetaxel |  |  |
| rs2228001 | TMEM43, XPC |  | docetaxel |  |  |
| rs2842951 | TPMT |  | docetaxel |  |  |
| rs1382368 | XRCC4 |  | docetaxel |  |  |
| rs2253316 | TGFBR3 |  | docetaxel |  |  |
| rs1883322 | PPARD |  | docetaxel; thalidomide |  | Prostatic Neoplasms |
| rs1799931 | NAT2 | Exon/NonSyn | docetaxel; thalidomide |  | Prostatic Neoplasms |
| rs3734254 | PPARD | 3' UTR | docetaxel; thalidomide |  | Prostatic Neoplasms |
| rs2238472 | ABCC6 | Exon/NonSyn | docetaxel; thalidomide |  | Prostatic Neoplasms |
| rs2016520 | PPARD | 5' UTR | docetaxel; thalidomide |  | Prostatic Neoplasms |
| rs1402467 | SULT1C4 | Exon/NonSyn | docetaxel; thalidomide |  | Prostatic Neoplasms |
| rs714368 | SLC22A16 |  | doxorubicin |  | Breast Neoplasms |
| rs8133052 | CBR3, RPS9P1 |  | doxorubicin | ANTHRACYCLINES AND RELATED SUBSTANCES | Breast Neoplasms; Drug Toxicity |
| rs20572 | CBR1, SETD4 |  | doxorubicin |  |  |
| rs9024 | CBR1, SETD4 |  | doxorubicin |  |  |
| rs4673 | CYBA |  | doxorubicin |  | Arrhythmias, Cardiac; Cardiomyopathies; Drug Toxicity; Lymphoma, Non-Hodgkin |
| rs6025 | F5 |  | drotrecogin alfa; tamoxifen | ESTROGENS; HORMONAL CONTRACEPTIVES FOR SYSTEMIC USE | Thromboembolism; venous thromboembolism |
| rs852977 | NR3C1 | Intron | escitalopram; nortriptyline |  | Depression |
| rs4986893 | CYP2C19 | Exon/NonSyn | esomeprazole |  | Gastroesophageal Reflux |
| rs1229984 | ADH1B | Exon/NonSyn | ethanol |  |  |
| rs975833 | ADH1A |  | ethanol |  |  |
| rs7378340 | SLC2A9 |  | etoposide |  | Drug Toxicity |
| rs1574091 | FGF12 |  | etoposide |  | Drug Toxicity |
| rs12498150 | SLC2A9 |  | etoposide |  | Drug Toxicity |
| rs2240920 | ITIH3 |  | etoposide |  | Drug Toxicity |
| rs2375699 | DNAJC6 |  | etoposide |  | Drug Toxicity |
| rs9994266 | SLC2A9 |  | etoposide |  | Drug Toxicity |
| rs7784162 | NUP205 |  | etoposide |  | Drug Toxicity |
| rs4580649 | SLC2A9 |  | etoposide |  | Drug Toxicity |
| rs7114978 | CNTN5 |  | etoposide |  | Drug Toxicity |
| rs186660 | BMP7 |  | etoposide |  | Drug Toxicity |
| rs456210 | ZNF663 |  | etoposide |  | Drug Toxicity |
| rs10825264 | PCDH15 |  | etoposide |  | Drug Toxicity |
| rs6449172 | SLC2A9 |  | etoposide |  | Drug Toxicity |
| rs1035339 | CNTN5 |  | etoposide |  | Drug Toxicity |
| rs1702176 | KCNN3 |  | etoposide |  | Drug Toxicity |
| rs12435920 | SLC24A4 |  | etoposide |  | Drug Toxicity |
| rs8180115 | STX18 |  | etoposide |  | Drug Toxicity |
| rs460703 | ZNF663 |  | etoposide |  | Drug Toxicity |
| rs1399180 | GATA3 | Intron | etoposide |  | Drug Toxicity |
| rs10983320 | ASTN2 |  | etoposide |  | Drug Toxicity |
| rs9993410 | SLC2A9 |  | etoposide |  | Drug Toxicity |
| rs12435905 | SLC24A4 |  | etoposide |  | Drug Toxicity |
| rs4919908 | C19orf20 |  | etoposide |  | Drug Toxicity |
| rs2134143 | KSR2 |  | etoposide |  | Drug Toxicity |
| rs17185870 | SLC2A9 |  | etoposide |  | Drug Toxicity |
| rs12963484 | FHOD3 |  | etoposide |  | Drug Toxicity |
| rs7672947 | SLC2A9 |  | etoposide |  | Drug Toxicity |
| rs6449174 | SLC2A9 |  | etoposide |  | Drug Toxicity |
| rs11023100 | SPON1 |  | etoposide |  | Drug Toxicity |
| rs7676733 | SLC2A9 |  | etoposide |  | Drug Toxicity |
| rs4621431 | SLC2A9 |  | etoposide |  | Drug Toxicity |
| rs7658170 | SLC2A9 |  | etoposide |  | Drug Toxicity |
| rs1604079 | KSR2 |  | etoposide |  | Drug Toxicity |
| rs4311316 | SLC2A9 |  | etoposide |  | Drug Toxicity |
| rs6843873 | SLC2A9 |  | etoposide |  | Drug Toxicity |
| rs403029 | GATA3 | NA | etoposide |  | Drug Toxicity |
| rs10018204 | SLC2A9 |  | etoposide |  | Drug Toxicity |
| rs6823361 | SLC2A9 |  | etoposide |  | Drug Toxicity |
| rs6815001 | SLC2A9 |  | etoposide |  | Drug Toxicity |
| rs6788148 | FGF12 |  | etoposide |  | Drug Toxicity |
| rs12277155 | CNTN5 |  | etoposide |  | Drug Toxicity |
| rs4339211 | SLC2A9 |  | etoposide |  | Drug Toxicity |
| rs9788969 | FAM38A |  | etoposide |  | Drug Toxicity |
| rs12282593 | CNTN5 |  | etoposide |  | Drug Toxicity |
| rs11222869 | NTM |  | etoposide |  | Drug Toxicity |
| rs4473653 | SLC2A9 |  | etoposide |  | Drug Toxicity |
| rs7376948 | SLC2A9 |  | etoposide |  | Drug Toxicity |
| rs12287931 | CNTN5 |  | etoposide |  | Drug Toxicity |
| rs6449171 | SLC2A9 |  | etoposide |  | Drug Toxicity |
| rs11201887 | GRID1 |  | etoposide |  | Drug Toxicity |
| rs7274072 | ZNF663 |  | etoposide |  | Drug Toxicity |
| rs737582 | CNTN5 |  | etoposide |  | Drug Toxicity |
| rs737583 | CNTN5 |  | etoposide |  | Drug Toxicity |
| rs6449157 | SLC2A9 |  | etoposide |  | Drug Toxicity |
| rs8042404 | FAM189A1 |  | etoposide |  | Drug Toxicity |
| rs1884679 | SLC24A4 |  | etoposide |  | Drug Toxicity |
| rs2407309 | CNTN5 |  | etoposide |  | Drug Toxicity |
| rs4078157 | GFRA2 |  | etoposide |  | Drug Toxicity |
| rs10481625 | PTPRD |  | etoposide |  | Drug Toxicity |
| rs2134142 | KSR2 |  | etoposide |  | Drug Toxicity |
| rs1079128 | SLC2A9 |  | etoposide |  | Drug Toxicity |
| rs7677710 | SLC2A9 |  | etoposide |  | Drug Toxicity |
| rs6590495 | CNTN5 |  | etoposide |  | Drug Toxicity |
| rs12573606 | GRID1 |  | etoposide |  | Drug Toxicity |
| rs2636802 | SLIT1 |  | etoposide |  | Drug Toxicity |
| rs3909276 | CNTN5 |  | etoposide |  | Drug Toxicity |
| rs11083241 | CDH2 |  | etoposide |  | Drug Toxicity |
| rs6449155 | SLC2A9 |  | etoposide |  | Drug Toxicity |
| rs2018643 | SLC2A9 |  | etoposide |  | Drug Toxicity |
| rs460869 | ZNF663 |  | etoposide |  | Drug Toxicity |
| rs446112 | ZNF663 |  | etoposide |  | Drug Toxicity |
| rs4519796 | SLC2A9 |  | etoposide |  | Drug Toxicity |
| rs13328050 | SLC2A9 |  | etoposide |  | Drug Toxicity |
| rs4919910 | C19orf20 |  | etoposide |  | Drug Toxicity |
| rs7331762 | HS6ST3 |  | etoposide |  | Drug Toxicity |
| rs2784917 | SLIT1 |  | etoposide |  | Drug Toxicity |
| rs7378305 | SLC2A9 |  | etoposide |  | Drug Toxicity |
| rs4312757 | SLC2A9 |  | etoposide |  | Drug Toxicity |
| rs7974925 | KSR2 |  | etoposide |  | Drug Toxicity |
| rs869191 | CAMK2A |  | etoposide |  | Drug Toxicity |
| rs1946468 | ADCY2 |  | etoposide |  | Drug Toxicity |
| rs6588131 | DNAJC6 |  | etoposide |  | Drug Toxicity |
| rs4702473 | ADCY2 |  | etoposide |  | Drug Toxicity |
| rs6880956 | ADCY2 |  | etoposide |  | Drug Toxicity |
| rs4562389 | PTPRD |  | etoposide |  | Drug Toxicity |
| rs4269885 | CNTN5 |  | etoposide |  | Drug Toxicity |
| rs6849729 | SLC2A9 |  | etoposide |  | Drug Toxicity |
| rs6449159 | SLC2A9 |  | etoposide |  | Drug Toxicity |
| rs4258342 | CNTN5 |  | etoposide |  | Drug Toxicity |
| rs6834893 | SLC2A9 |  | etoposide |  | Drug Toxicity |
| rs6814664 | SLC2A9 |  | etoposide |  | Drug Toxicity |
| rs2914295 | ADCY2 |  | etoposide |  | Drug Toxicity |
| rs4455410 | SLC2A9 |  | etoposide |  | Drug Toxicity |
| rs2303500 | FBN1 |  | etoposide |  | Drug Toxicity |
| rs888597 | CNTN5 |  | etoposide |  | Drug Toxicity |
| rs17185835 | SLC2A9 |  | etoposide |  | Drug Toxicity |
| rs6449201 | SLC2A9 |  | etoposide |  | Drug Toxicity |
| rs1833489 | CNTN5 |  | etoposide |  | Drug Toxicity |
| rs12498956 | SLC2A9 |  | etoposide |  | Drug Toxicity |
| rs12828172 | KSR2 |  | etoposide |  | Drug Toxicity |
| rs7125241 | CNTN5 |  | etoposide |  | Drug Toxicity |
| rs2636797 | SLIT1 |  | etoposide |  | Drug Toxicity |
| rs9594738 | FABP3P2 |  | etoposide |  | Drug Toxicity |
| rs7125266 | CNTN5 |  | etoposide |  | Drug Toxicity |
| rs6839490 | SLC2A9 |  | etoposide |  | Drug Toxicity |
| rs2607839 | GRID1 |  | etoposide |  | Drug Toxicity |
| rs12289401 | CNTN5 |  | etoposide |  | Drug Toxicity |
| rs9533089 | FABP3P2 |  | etoposide |  | Drug Toxicity |
| rs6844316 | SLC2A9 |  | etoposide |  | Drug Toxicity |
| rs16905691 | PCDH15 |  | etoposide |  | Drug Toxicity |
| rs4604727 | DNM3 |  | etoposide |  | Drug Toxicity |
| rs1111599 | KSR2 |  | etoposide |  | Drug Toxicity |
| rs803917 | ASTN2 |  | etoposide |  | Drug Toxicity |
| rs6784434 | FGF12 |  | etoposide |  | Drug Toxicity |
| rs12068388 | UBXN11 |  | etoposide |  | Drug Toxicity |
| rs10774929 | KSR2 |  | etoposide |  | Drug Toxicity |
| rs1377580 | CNTN5 |  | etoposide |  | Drug Toxicity |
| rs1846681 | ADCY2 |  | etoposide |  | Drug Toxicity |
| rs12273744 | CNTN5 |  | etoposide |  | Drug Toxicity |
| rs6852441 | SLC2A9 |  | etoposide |  | Drug Toxicity |
| rs13174252 | ADCY2 |  | etoposide |  | Drug Toxicity |
| rs4767551 | KSR2 |  | etoposide |  | Drug Toxicity |
| rs9299075 | PTPRD |  | etoposide |  | Drug Toxicity |
| rs13103879 | SLC2A9 |  | etoposide |  | Drug Toxicity |
| rs6449178 | SLC2A9 |  | etoposide |  | Drug Toxicity |
| rs9533090 | FABP3P2 |  | etoposide |  | Drug Toxicity |
| rs6590489 | CNTN5 |  | etoposide |  | Drug Toxicity |
| rs1846644 | KSR2 |  | etoposide |  | Drug Toxicity |
| rs12674093 | BBS9 |  | etoposide |  | Drug Toxicity |
| rs7686538 | SLC2A9 |  | etoposide |  | Drug Toxicity |
| rs6449176 | SLC2A9 |  | etoposide |  | Drug Toxicity |
| rs10038196 | ADCY2 |  | etoposide |  | Drug Toxicity |
| rs11222291 | CNTN5 |  | etoposide |  | Drug Toxicity |
| rs320 | LPL |  | fenofibrate |  | Hyperlipidemias; Hypertriglyceridemia |
| rs10937275 | ST6GAL1 |  | flucloxacillin |  | Drug Toxicity; Liver Diseases |
| rs1801159 | DPYD | Exon/NonSyn | fluorouracil |  | Nausea; Vomiting |
| rs1801265 | DPYD | Exon/NonSyn | fluorouracil |  | Nausea; Vomiting |
| rs10042486 | HTR1A |  | fluvoxamine; paroxetine |  | Depression |
| rs3775289 | DCK | Intron | gemcitabine |  |  |
| rs2242047 | SLC28A1 | Exon/NonSyn | gemcitabine |  |  |
| rs1060896 | SLC28A2 | Exon/NonSyn | gemcitabine |  |  |
| rs909706 | DTNBP1 |  | haloperidol |  | Schizophrenia |
| rs4686799 | KNG1 | Intron | hydrochlorothiazide | ANTIHYPERTENSIVES |  |
| rs1800169 | CNTF, ZFP91, ZFP91-CNTF |  | iloperidone |  |  |
| rs1061622 | TNFRSF1B |  | infliximab |  | Arthritis, Rheumatoid |
| rs4796793 | STAT3 |  | interferon alfacon-1 |  | Carcinoma, Renal Cell |
| rs2622604 | ABCG2 | Intron | irinotecan |  | Anemia; Drug Toxicity; Leukopenia; Neoplasms; Neutropenia; Thrombocytopenia |
| rs7586110 | UGT1A10, UGT1A6, UGT1A7, UGT1A8, UGT1A9 | Intron, Intron, Intron, Intron, Intron | irinotecan |  | Anemia |
| rs17868323 | UGT1A10, UGT1A6, UGT1A7, UGT1A8, UGT1A9 | Intron, NA, Exon/NonSyn, Intron, Intron | irinotecan |  | Diarrhea; Thrombocytopenia |
| rs4148323 | UGT1A10, UGT1A3, UGT1A4, UGT1A5, UGT1A6, UGT1A7, UGT1A8, UGT1A9, UGT1A1 | Intron, Intron, Intron, Intron, Intron, Intron, Intron, Intron, Exon/NonSyn | irinotecan |  | Gilbert's syndrome |
| rs10918594 | NOS1AP |  | isradipine; verapamil |  |  |
| rs4646 | CYP19A1 | 3' UTR | letrozole |  | Breast Neoplasms |
| rs2284017 | CACNG2 |  | lithium |  | Bipolar Disorder |
| rs2284018 | CACNG2 |  | lithium |  | Bipolar Disorder |
| rs5750285 | CACNG2 |  | lithium |  | Bipolar Disorder |
| rs622342 | SLC22A1 |  | metformin |  |  |
| rs2070995 | KCNJ6 |  | methadone |  |  |
| rs2619539 | DTNBP1 |  | methamphetamine |  | Bipolar Disorder; Psychotic Disorders; Schizophrenia |
| rs2076369 | PICK1 |  | methamphetamine |  | Psychotic Disorders; Substance-Related Disorders |
| rs3026682 | PICK1 |  | methamphetamine |  |  |
| rs2853539 | TYMS, C18orf56 |  | methotrexate |  | Arthritis, Rheumatoid |
| rs246240 | ABCC1 |  | methotrexate |  |  |
| rs35592 | ABCC1 |  | methotrexate |  |  |
| rs4905865 | CCDC85C |  | methotrexate |  | Neoplasm, Residual; Precursor Cell Lymphoblastic Leukemia-Lymphoma |
| rs3761422 | ADORA2A |  | methotrexate |  | Arthritis, Rheumatoid; Drug Toxicity |
| rs2236624 | ADORA2A |  | methotrexate |  | Arthritis, Rheumatoid; Drug Toxicity |
| rs4846051 | MTHFR | Exon/Syn | methotrexate |  | Arthritis, Rheumatoid |
| rs5760410 | CYTSA |  | methotrexate |  | Arthritis, Rheumatoid; Drug Toxicity |
| rs2298383 | ADORA2A |  | methotrexate |  | Arthritis, Rheumatoid; Drug Toxicity |
| rs10994982 | ARID5B | Intron | methotrexate |  | Precursor Cell Lymphoblastic Leukemia-Lymphoma |
| rs2267076 | ADORA2A |  | methotrexate |  | Arthritis, Rheumatoid; Drug Toxicity |
| rs1800909 | GGH | Exon/NonSyn | methotrexate |  | Precursor Cell Lymphoblastic Leukemia-Lymphoma |
| rs11160533 | CCDC85C |  | methotrexate |  | Neoplasm, Residual; Precursor Cell Lymphoblastic Leukemia-Lymphoma |
| rs11045879 | SLCO1B1 | Intron | methotrexate |  | Precursor Cell Lymphoblastic Leukemia-Lymphoma |
| rs3758149 | GGH | NA | methotrexate |  | Arthritis, Rheumatoid |
| rs4149081 | SLCO1B1 | Intron | methotrexate |  | Precursor Cell Lymphoblastic Leukemia-Lymphoma |
| rs1051266 | SLC19A1 | Exon/NonSyn | methotrexate |  | Arthritis, Rheumatoid |
| rs1065852 | CYP2D6 | Exon/NonSyn | metoprolol |  |  |
| rs1464603 | NR1I2 | Intron | midazolam |  |  |
| rs1464602 | NR1I2 |  | midazolam |  |  |
| rs2115819 | ALOX5 | Intron | montelukast |  | Asthma |
| rs2660845 | LTA4H | Intron | montelukast |  | Asthma |
| rs5370 | EDN1 |  | muraglitazar |  | Diabetes Mellitus; Edema; Hyperlipidemias |
| rs2278293 | IMPDH1 | Intron | mycophenolate mofetil |  |  |
| rs2278294 | IMPDH1 | Intron | mycophenolate mofetil |  |  |
| rs4149117 | SLCO1B3 | Exon/NonSyn | mycophenolate mofetil; mycophenolic acid; sirolimus; tacrolimus |  | Organ Transplantation |
| rs578776 | CHRNA5, CHRNA3 |  | nicotine |  | Tobacco Use Disorder |
| rs4423615 | GRB14 |  | nicotine |  |  |
| rs4725563 | TBXAS1 |  | nicotine |  |  |
| rs680244 | CHRNA5 |  | nicotine |  | Tobacco Use Disorder |
| rs1044396 | CHRNA4 |  | nicotine |  | Tobacco Use Disorder |
| rs4509385 | MSRA |  | nicotine |  |  |
| rs6997956 | KHDRBS3 |  | nicotine |  |  |
| rs6463462 | UPP1 |  | nicotine |  |  |
| rs1451371 | DDC |  | nicotine |  | Tobacco Use Disorder |
| rs569207 | CHRNA5 |  | nicotine |  | Tobacco Use Disorder |
| rs4141130 | DCP1B |  | nicotine |  |  |
| rs2236196 | CHRNA4 |  | nicotine |  | Tobacco Use Disorder |
| rs186715 | EMR1 |  | nicotine |  |  |
| rs1599903 | MYRIP |  | nicotine |  |  |
| rs2072661 | CHRNB2 |  | nicotine |  | Tobacco Use Disorder |
| rs5771934 | FAM19A5 |  | nicotine |  |  |
| rs3764213 | NIPA2 |  | nicotine |  |  |
| rs9554711 | TMTC4 |  | nicotine |  |  |
| rs10868236 | NTRK2 |  | nicotine |  |  |
| rs921451 | DDC |  | nicotine |  | Tobacco Use Disorder |
| rs3735273 | DDC |  | nicotine |  | Tobacco Use Disorder |
| rs3745009 | SLC14A2 |  | nifedipine |  | Hypertension |
| rs1801131 | MTHFR | Exon/NonSyn | nitrous oxide |  |  |
| rs2500535 | UST |  | nortriptyline |  |  |
| rs4731426 | LEP |  | olanzapine |  | Schizophrenia |
| rs518147 | HTR2C |  | olanzapine |  | Schizophrenia; Weight gain |
| rs762551 | CYP1A2 |  | olanzapine |  |  |
| rs3813929 | HTR2C |  | olanzapine |  | Weight gain |
| rs7997012 | HTR2A |  | olanzapine |  |  |
| rs1799732 | DRD2 |  | olanzapine; risperidone |  | Schizophrenia |
| rs1056836 | CYP1B1 | Exon/NonSyn | paclitaxel |  | Breast Neoplasms |
| rs1113129 | CYP2C8 |  | paclitaxel |  | Drug Toxicity; Neurotoxicity Syndromes |
| rs1934951 | CYP2C8 |  | pamidronate; zoledronate |  | Osteonecrosis |
| rs12050217 | BDKRB1 |  | perindopril | ACE INHIBITORS, PLAIN | Coronary Artery Disease |
| rs2842030 | RGS4 |  | perphenazine; quetiapine; risperidone; ziprasidone |  | Schizophrenia |
| rs1051740 | EPHX1 | Exon/NonSyn | phenytoin |  | Craniofacial Abnormalities |
| rs11615 | ERCC1 |  | platinum |  | Carcinoma, Non-Small-Cell Lung |
| rs3212986 | CD3EAP, PPP1R13L, ERCC1 |  | platinum |  | Carcinoma, Non-Small-Cell Lung |
| rs4149015 | SLCO1B1 |  | pravastatin |  |  |
| rs12654264 | HMGCR |  | pravastatin; simvastatin | HMG COA REDUCTASE INHIBITORS | Colorectal Neoplasms |
| rs2032582 | ABCB1 | Exon/NonSyn | rhodamine 123; rifampin; verapamil |  |  |
| rs1045642 | ABCB1 | Exon/Syn | rifampin; verapamil |  |  |
| rs2661319 | RGS4 |  | risperidone |  | Schizophrenia |
| rs724226 | GRM3 |  | risperidone |  | Schizophrenia |
| rs167771 | DRD3 |  | risperidone |  | Bipolar Disorder; Dyskinesia, Drug-Induced; Dystonia; Hypokinesia; Muscle Rigidity; Schizophrenia; Tremor |
| rs7799039 | LEP |  | risperidone |  |  |
| rs2494732 | AKT1 |  | risperidone |  | Schizophrenia |
| rs1457266 | NEFM |  | risperidone | ANTIPSYCHOTICS | Schizophrenia |
| rs3813928 | HTR2C |  | risperidone |  | Autistic Disorder |
| rs5128 | APOA4, APOA1, APOC3 | Intron, Intron, 3' UTR | ritonavir |  | HIV; HIV Infections; Hyperlipidemias |
| rs2231142 | ABCG2 | Exon/NonSyn | rosuvastatin |  |  |
| rs255100 | CRHR2 | Intron | salbutamol | SELECTIVE BETA-2-ADRENORECEPTOR AGONISTS | Asthma |
| rs2267715 | CRHR2 | Intron | salbutamol | SELECTIVE BETA-2-ADRENORECEPTOR AGONISTS | Asthma |
| rs776746 | CYP3A5, CYP3A | Intron, NA | tacrolimus |  | Organ Transplantation; Transplantation |
| rs16947 | CYP2D6 | Exon/NonSyn | tamoxifen |  | Breast Neoplasms |
| rs11653 | OPN1SW, CALU |  | warfarin |  |  |
| rs216013 | CACNA1C |  | warfarin |  |  |
| rs10871454 | STX4 |  | warfarin |  |  |
| rs4917639 | CYP2C9 |  | warfarin |  |  |
| rs2108622 | CYP4F2 | Exon/NonSyn | warfarin |  |  |
| rs2292566 | EPHX1 | Exon/Syn | warfarin |  |  |
| rs4086116 | CYP2C9 |  | warfarin |  |  |
| rs2260863 | EPHX1 |  | warfarin |  |  |
| rs8050894 | VKORC1 | Intron | warfarin |  |  |
| rs9923231 | VKORC1 | NA | warfarin |  |  |
