# Supplementary material for: Systematic Pharmacogenomics Analysis of a Malay Whole Genome: Proof of Concept for Personalized Medicine
Source: PLoS One. 2013 Aug 23;8(8):e71554. doi: 10.1371/journal.pone.0071554 (PMC3751891; doi:10.1371/journal.pone.0071554)
Supplement: Table S5 — List of drug pathway genes harboring deleterious mutations and the drugs affected. (DOCX) [file pone.0071554.s008.docx]

**Table S5: List of drug pathway genes harboring deleterious mutations and the drugs affected**

| dbSNP ID | aa mutation | Gene | Category | Drug name | Drug class |
| --- | --- | --- | --- | --- | --- |
| rs2300455 | A651T | ACACB | Target | Biotin | Dietary supplement |
|  |  | ACACB | Target | Adenine | Dietary supplement |
|  |  | ACACB | Target | Soraphen A | Rheumatology |
|  |  | ACACB | Target | 2-[4-(2,4-Dichlorophenoxy)Phenoxy]Propanoic Acid | Under trial |
|  |  | ACACB | Target | (2S)-2-(4-{[3-CHLORO-5-(TRIFLUOROMETHYL)PYRIDIN-2-YL]OXY}PHENOXY)PROPANOIC ACID | Under trial |
| rs2240154 | T157M | GRIN3B | Target | L-Glutamic Acid | Dietary supplement |
|  |  | GRIN3B | Target | Glycine | Dietary supplement |
|  |  | GRIN3B | Target | Halothane | Neurology |
|  |  | GRIN3B | Target | Orphenadrine | Psychiatry |
|  |  | GRIN3B | Target | Tenocyclidine | Neurology |
| Novel | Y250N | PDE8A | Target | Ketotifen | Allergy |
|  |  | PDE8A | Target | 3-ISOBUTYL-1-METHYLXANTHINE | Under trial |
| Novel | D58G | NDUFA7 | Target | NADH | Dietary supplement |
| Novel | T430K | FGA | Target | Alteplase | Hematology |
|  |  | FGA | Target | Reteplase | Hematology |
|  |  | FGA | Target | Anistreplase | Hematology |
|  |  | FGA | Target | Tenecteplase | Hematology |
|  |  | FGA | Target | Sucralfate | Gastroenterology |
|  |  | FGA | Target | Alpha-D-Mannose | Dietary supplement |
| rs7962217 | G2705R | VWF | Target | Antihemophilic Factor | Hematology |
| rs4646422 | G45D | CYP1A1 | Enzyme | Vitamin A | Dietary supplement |
|  |  | CYP1A1 | Enzyme | Cholecalciferol | Dietary supplement |
|  |  | CYP1A1 | Enzyme | Menadione | Hematology |
|  |  | CYP1A1 | Enzyme | Fluvoxamine | Psychiatry |
|  |  | CYP1A1 | Enzyme | Nicotine | Neurology |
|  |  | CYP1A1 | Enzyme | Bortezomib | Oncology |
|  |  | CYP1A1 | Enzyme | Troglitazone | Metabolic and Endocrinology |
|  |  | CYP1A1 | Enzyme | Caffeine | Pulmonary |
|  |  | CYP1A1 | Enzyme | Sildenafil | Neurology |
|  |  | CYP1A1 | Enzyme | Ticlopidine | Hematology |
|  |  | CYP1A1 | Enzyme | Theophylline | Pulmonary |
|  |  | CYP1A1 | Enzyme | Acetaminophen | Analgesics |
|  |  | CYP1A1 | Enzyme | Nitroprusside | Cardiovascular |
|  |  | CYP1A1 | Enzyme | Omeprazole | Gastroenterology |
|  |  | CYP1A1 | Enzyme | Chlorzoxazone | Musculoskeletal |
|  |  | CYP1A1 | Enzyme | Clozapine | Psychiatry |
|  |  | CYP1A1 | Enzyme | Amlodipine | Cardiovascular |
|  |  | CYP1A1 | Enzyme | Progesterone | Steroidal |
|  |  | CYP1A1 | Enzyme | Lansoprazole | Gastroenterology |
|  |  | CYP1A1 | Enzyme | Prazosin | Cardiovascular |
|  |  | CYP1A1 | Enzyme | Quinine | Antiinfectives |
|  |  | CYP1A1 | Enzyme | Dronabinol | Gastroenterology |
|  |  | CYP1A1 | Enzyme | Flutamide | Oncology |
|  |  | CYP1A1 | Enzyme | Haloperidol | Psychiatry |
|  |  | CYP1A1 | Enzyme | Albendazole | Antiinfectives |
|  |  | CYP1A1 | Enzyme | Oxaliplatin | Oncology |
|  |  | CYP1A1 | Enzyme | Erlotinib | Oncology |
|  |  | CYP1A1 | Enzyme | Toremifene | Oncology |
|  |  | CYP1A1 | Enzyme | Methoxsalen | Dermatology and Dental |
|  |  | CYP1A1 | Enzyme | Cinnarizine | Gastroenterology |
|  |  | CYP1A1 | Enzyme | Propranolol | Cardiovascular |
|  |  | CYP1A1 | Enzyme | Clonidine | Cardiovascular |
|  |  | CYP1A1 | Enzyme | Diclofenac | Analgesics |
|  |  | CYP1A1 | Enzyme | Sulindac | Analgesics |
|  |  | CYP1A1 | Enzyme | Chloroquine | Antiinfectives |
|  |  | CYP1A1 | Enzyme | Amodiaquine | Antiinfectives |
|  |  | CYP1A1 | Enzyme | Testosterone | Androgens |
|  |  | CYP1A1 | Enzyme | Clofibrate | Metabolic and Endocrinology |
|  |  | CYP1A1 | Enzyme | Mebendazole | Antiinfectives |
|  |  | CYP1A1 | Enzyme | Estrone | Steroidal |
|  |  | CYP1A1 | Enzyme | Tamoxifen | Oncology |
|  |  | CYP1A1 | Enzyme | Warfarin | Oncology |
|  |  | CYP1A1 | Enzyme | Daunorubicin | Oncology |
|  |  | CYP1A1 | Enzyme | Thiabendazole | Antiinfectives |
|  |  | CYP1A1 | Enzyme | Pentamidine | Antiinfectives |
|  |  | CYP1A1 | Enzyme | Riluzole | Musculoskeletal |
|  |  | CYP1A1 | Enzyme | Tretinoin | Dermatology and Dental |
|  |  | CYP1A1 | Enzyme | Estradiol | Steroidal |
|  |  | CYP1A1 | Enzyme | Dicyclomine | Gastroenterology |
|  |  | CYP1A1 | Enzyme | Propofol | Neurology |
|  |  | CYP1A1 | Enzyme | Dacarbazine | Oncology |
|  |  | CYP1A1 | Enzyme | Clomifene | Metabolic and Endocrinology |
|  |  | CYP1A1 | Enzyme | Granisetron | Gastroenterology |
|  |  | CYP1A1 | Enzyme | Ethanol | Neurology |
|  |  | CYP1A1 | Enzyme | Quinidine | Cardiovascular |
|  |  | CYP1A1 | Enzyme | Azelastine | Allergy |
|  |  | CYP1A1 | Enzyme | Clobetasol | Steroidal |
|  |  | CYP1A1 | Enzyme | Ketoconazole | Antiinfectives |
|  |  | CYP1A1 | Enzyme | Thalidomide | Oncology |
|  |  | CYP1A1 | Enzyme | Norfloxacin | Antiinfectives |
|  |  | CYP1A1 | Enzyme | Isoproterenol | Cardiovascular |
|  |  | CYP1A1 | Enzyme | Melatonin | Neurology |
|  |  | CYP1A1 | Enzyme | Primaquine | Antiinfectives |
|  |  | CYP1A1 | Enzyme | Ouabain | Cardiovascular |
|  |  | CYP1A1 | Enzyme | Fluvastatin | Metabolic and Endocrinology |
|  |  | CYP1A1 | Enzyme | Nifedipine | Cardiovascular |
|  |  | CYP1A1 | Enzyme | Amiodarone | Cardiovascular |
|  |  | CYP1A1 | Enzyme | Rabeprazole | Gastroenterology |
|  |  | CYP1A1 | Enzyme | Carvedilol | Cardiovascular |
|  |  | CYP1A1 | Enzyme | Itraconazole | Antiinfectives |
|  |  | CYP1A1 | Enzyme | Arsenic trioxide | Oncology |
|  |  | CYP1A1 | Enzyme | Dexamethasone | Steroidal |
|  |  | CYP1A1 | Enzyme | Dasatinib | Oncology |
|  |  | CYP1A1 | Enzyme | Mibefradil | Cardiovascular |
|  |  | CYP1A1 | Enzyme | Bezafibrate | Metabolic and Endocrinology |
|  |  | CYP1A1 | Enzyme | Clenbuterol | Pulmonary |
|  |  | CYP1A1 | Enzyme | Resveratrol | Antiinfectives |
|  |  | CYP1A1 | Enzyme | Phenacetin | Analgesics |
|  |  | CYP1A1 | Enzyme | Debrisoquin | Cardiovascular |
|  |  | CYP1A1 | Enzyme | Flunarizine | Neurology |
| rs235330 | Q354H | ITGB2 | Target | Simvastatin | Metabolic and Endocrinology |
| rs2230287 | A89T | GSN | Target | Latrunculin A | Under trial/oncology |
| rs10891314 | D346N | DLAT | Target | NADH | Dietary supplement |
|  |  | DLAT | Target | Radicicol | Antiinfectives |
|  |  | DLAT | Target | Dihydrolipoic Acid | Dietary supplement |
| rs6180 | I544L | GHR | Target | Somatropin recombinant | Metabolic and Endocrinology |
|  |  | GHR | Target | Pegvisomant | Metabolic and Endocrinology |
| rs2228976 | A303S | PYGB | Target | Pyridoxal Phosphate | Dietary supplement |
| rs8191754 | L252V | IGF2R | Target | Mecasermin | Metabolic and Endocrinology |
|  |  | IGF2R | Target | Alpha-D-Mannose-6-Phosphate | Under trial |
|  |  | IGF2R | Target | Alpha-D-Mannose-6-Phosphate | Dietary supplement |
|  |  | IGF2R | Target | Alpha-D-Mannose | Dietary supplement |
| rs1933437 | T227M | FLT3 | Target | Sorafenib | Oncology |
|  |  | FLT3 | Target | Sunitinib | Oncology |
| Novel | R38H | ADH7 | Enzyme | NADH | Dietary supplement |
| Novel | R38H | ADH7 | Target | NADH | Dietary supplement |
|  |  | ADH7 | Target | Nicotinamide-Adenine-Dinucleotide | Dietary supplement |
|  |  | ADH7 | Target | Nicotinamide-Adenine-Dinucleotide | Under trial |
| Novel | C154W | AKR1C3 | Target | NADH | Dietary supplement |
|  |  | AKR1C3 | Target | Bimatoprost | Metabolic and Endocrinology |
|  |  | AKR1C3 | Target | 4-Androstenedione | Steroidal |
|  |  | AKR1C3 | Target | Rutin | Cardiovascular |
|  |  | AKR1C3 | Target | (5e,13e)-9,15-Dihydroxy-11-Oxoprosta-5,13-Dien-1-Oicacid | Under trial |
|  |  | AKR1C3 | Target | Flufenamic Acid | Analgesics |
|  |  | AKR1C3 | Target | 2'-Monophosphoadenosine 5'-Diphosphoribose | Under trial |
|  |  | AKR1C3 | Target | 3-CARBOXAMIDO-1,3,5(10)-ESTRATRIEN-17(R)-SPIRO-2'(5',5'-DIMETHYL-6'OXO)TETRAHYDROPYRAN | Under trial |
| rs17856219 | R276W | ALDH3B2 | Target | NADH | Dietary supplement |
| Novel | R101C | B2M | Target | 3-Indolebutyric Acid | Under trial |
|  |  | B2M | Target | N-Formylmethionine | Dietary supplement |
|  |  | B2M | Target | N-Formylmethionine | Others |
| rs897471 | A1503V | HSPG2 | Target | Palifermin | Antimuscotic agents |
| rs4667591 | I4210L | LRP2 | Target | Urokinase | Thrombolytic agents |
|  |  | LRP2 | Target | Insulin recombinant | Metabolic and Endocrinology |
|  |  | LRP2 | Target | Insulin, porcine | Metabolic and Endocrinology |
|  |  | LRP2 | Target | Gentamicin | Antiinfectives |
| rs569108 | E237G | MS4A2 | Target | Omalizumab | Allergy |
| Novel | R128H | CHIT1 | Target | N-Acetyl-D-Allosamine | Under trial |
|  |  | CHIT1 | Target | 2-(Acetylamino)-2-Deoxy-6-O-Methyl-Alpha-D-Allopyranose | Under trial |
|  |  | CHIT1 | Target | Argifin | Under trial |
|  |  | CHIT1 | Target | Argadin | Under trial |
|  |  | CHIT1 | Target | Allosamizoline | Under trial |
|  |  | KCNJ12 | Target | Dofetilide | Cardiovascular |
| rs1201559 | P516L | SLC22A10 | Transporter | Conjugated Estrogens | Steroidal |
|  |  | SLC22A10 | Transporter | Salicyclic acid | Analgesics |
|  |  | SLC22A10 | Transporter | Probenecid | Rheumatology |
| rs17868387 | Y251C | TRPM8 | Target | Menthol | Dermatology and Dental |
| rs1128431 | I566V | EFTUD1 | Target | S-(Methylmercury)-L-Cysteine | Under trial |
|  |  | EFTUD1 | Target | Guanosine-5'-Diphosphate | Under trial |
|  |  | EFTUD1 | Target | (3ALPHA,5BETA,12ALPHA)-3,12-DIHYDROXYCHOLAN-24-OIC ACID | Under trial |
| rs35762223 | H195R | GLT6D1 | Target | Beta-D-Glucose | Dietary supplement |
|  |  | GLT6D1 | Target | Uridine-5'-Diphosphate | Under trial |
|  |  | GLT6D1 | Target | Uridine-5'-Monophosphate | Under trial |
|  |  | GLT6D1 | Target | Uridine-5'-Monophosphate Glucopyranosyl-Monophosphateester | Under trial |
|  |  | GLT6D1 | Target | Uridine-5'-Monophosphate Glucopyranosyl-Monophosphateester | Under trial |
|  |  | GLT6D1 | Target | Lactose | Dietary supplement |
|  |  | ABCA6 | Target | Adenosine-5'-Diphosphate | Under trial |
| Novel | L441M, I448M | ABCC2 | Target | Adenosine triphosphate | Dietary supplement |
|  |  | ABCC2 | Target | Sulfinpyrazone | Rheumatology |
|  |  | ABCC2 | Transporter | Vasopressin | Metabolic and Endocrinology |
|  |  | ABCC2 | Transporter | Cyclosporine | Analgesics |
|  |  | ABCC2 | Transporter | Tetrahydrofolic acid | Dietary supplement |
|  |  | ABCC2 | Transporter | Tetrahydrofolic acid | Dietary supplement |
|  |  | ABCC2 | Transporter | Glutathione | Dietary supplement |
|  |  | ABCC2 | Transporter | Pravastatin | Metabolic and Endocrinology |
|  |  | ABCC2 | Transporter | Reserpine | Cardiovascular |
|  |  | ABCC2 | Transporter | Indinavir | Antiinfectives |
|  |  | ABCC2 | Transporter | Phenytoin | Cardiovascular |
|  |  | ABCC2 | Transporter | Clotrimazole | Antiinfectives |
|  |  | ABCC2 | Transporter | Olmesartan | Cardiovascular |
|  |  | ABCC2 | Transporter | Conjugated Estrogens | Steroidal |
|  |  | ABCC2 | Transporter | Indomethacin | Analgesics |
|  |  | ABCC2 | Transporter | Aminohippurate | Diagnostic agent |
|  |  | ABCC2 | Transporter | Grepafloxacin | Antiinfectives |
|  |  | ABCC2 | Transporter | Sorafenib | Oncology |
|  |  | ABCC2 | Transporter | Spironolactone | Reproductive and urologic |
|  |  | ABCC2 | Transporter | Streptozocin | Oncology |
|  |  | ABCC2 | Transporter | Cerivastatin | Metabolic and Endocrinology |
|  |  | ABCC2 | Transporter | Ritonavir | Antiinfectives |
|  |  | ABCC2 | Transporter | Cisplatin | Oncology |
|  |  | ABCC2 | Transporter | Vincristine | Oncology |
|  |  | ABCC2 | Transporter | Methotrexate | Oncology |
|  |  | ABCC2 | Transporter | Vinblastine | Oncology |
|  |  | ABCC2 | Transporter | Ivermectin | Antiinfectives |
|  |  | ABCC2 | Transporter | Leucovorin | Dietary supplement |
|  |  | ABCC2 | Transporter | Verapamil | Cardiovascular |
|  |  | ABCC2 | Transporter | Daunorubicin | Oncology |
|  |  | ABCC2 | Transporter | Furosemide | Reproductive and urologic |
|  |  | ABCC2 | Transporter | Etoposide | Oncology |
|  |  | ABCC2 | Transporter | Sulfasalazine | Gastroenterology/Rheumatology |
|  |  | ABCC2 | Transporter | Eprosartan | Cardiovascular |
|  |  | ABCC2 | Transporter | Quinidine | Cardiovascular |
|  |  | ABCC2 | Transporter | Norgestimate | Steroidal |
|  |  | ABCC2 | Transporter | Telmisartan | Cardiovascular |
|  |  | ABCC2 | Transporter | Ezetimibe | Metabolic and Endocrinology |
|  |  | ABCC2 | Transporter | Ethinyl Estradiol | Steroidal |
|  |  | ABCC2 | Transporter | Ethinyl Estradiol | Steroidal |
|  |  | ABCC2 | Transporter | Lomefloxacin | Antiinfectives |
|  |  | ABCC2 | Transporter | Glyburide | Cardiovascular |
|  |  | ABCC2 | Transporter | Probenecid | Rheumatology |
|  |  | ABCC2 | Transporter | Rifampin | Antiinfectives |
|  |  | ABCC2 | Transporter | Nifedipine | Cardiovascular |
|  |  | ABCC2 | Transporter | Sulfinpyrazone | Rheumatology |
|  |  | ABCC2 | Transporter | Ofloxacin | Antiinfectives |
|  |  | ABCC2 | Transporter | Arsenic trioxide | Oncology |
|  |  | ABCC2 | Transporter | Phenobarbital | Neurology |
|  |  | ABCC2 | Transporter | Levetiracetam | Psychiatry |
|  |  | ABCC2 | Transporter | Sparfloxacin | Antiinfectives |
|  |  | ABCC2 | Transporter | Saquinavir | Antiinfectives |
|  |  | ABCC2 | Transporter | Dexamethasone | Steroidal |
|  |  | ABCC2 | Transporter | Sunitinib | Oncology |
|  |  | ABCC2 | Transporter | Pranlukast | Pulmonary |
|  |  | ABCC2 | Transporter | Ursodeoxycholic acid | Under trial |
|  |  | ABCC2 | Transporter | Ursodeoxycholic acid | Gastroenterology |
|  |  | ABCC2 | Transporter | Hyperforin | Others |
|  |  | ABCC2 | Transporter | Cholic Acid | Under trial |
|  |  | ABCC2 | Transporter | Fusidic Acid | Antiinfectives |
|  |  | ABCC2 | Transporter | (1,10 Phenanthroline)-(Tri-Carbon Monoxide) Rhenium (I) | Under trial |
|  |  | ABCC2 | Transporter | Quercetin | Under trial |
|  |  | ABCC2 | Transporter | Taurocholic Acid | Under trial |
|  |  | ABCC2 | Transporter | Taurocholic Acid | Steroidal |
|  |  | ABCC2 | Transporter | 5-methyltetrahydrofolate | Under trial |
| rs2293925 | R525W | TOP1MT | Transporter | Irinotecan | Oncology |
|  |  | TOP1MT | Transporter | Topotecan | Oncology |
| rs17822931 | G180R | ABCC11 | Transporter | Folic Acid | Dietary supplement |
|  |  | ABCC11 | Transporter | Conjugated Estrogens | Steroidal |
|  |  | ABCC11 | Transporter | Indomethacin | Analgesics |
|  |  | ABCC11 | Transporter | Methotrexate | Oncology |
|  |  | ABCC11 | Transporter | Probenecid | Rheumatology |
|  |  | ABCC11 | Transporter | Taurocholic Acid | Under trial |
|  |  | ABCC11 | Transporter | Taurocholic Acid | Steroidal |
| rs3809529 | P30L | LDHAL6B | Target | NADH | Dietary supplement |
| Novel | E367G | ADAM33 | Target | Alpha-D-Mannose | Dietary supplement |
| Novel | R131H | TPK1 | Target | Thiamine | Dietary supplement |
|  |  | TPK1 | Target | Pyrithiamine Pyrophosphate | Dietary supplement |
|  |  | TPK1 | Target | Pyrithiamine Pyrophosphate | Under trial |
| rs1801394 | I49M | MTRR | Target | Cyanocobalamin | Dietary supplement |
|  |  | MTRR | Target | L-Methionine | Dietary supplement |
|  |  | MTRR | Target | Hydroxocobalamin | Dietary supplement |
| rs117286870 | V134M | GPR44 | Target | Indomethacin | Analgesics |
|  |  | GPR44 | Target | Sulindac | Analgesics |
|  |  | GPR44 | Target | Zomepirac | Analgesics |
