# Supplementary material for: Systematic Pharmacogenomics Analysis of a Malay Whole Genome: Proof of Concept for Personalized Medicine
Source: PLoS One. 2013 Aug 23;8(8):e71554. doi: 10.1371/journal.pone.0071554 (PMC3751891; doi:10.1371/journal.pone.0071554)
Supplement: Table S6 — List of drugs (PharmGKB) implicated with the genetic variation of the Malay individual. (DOCX) [file pone.0071554.s009.docx]

**Table S6: List of drugs (PharmGKB) implicated with the genetic variation of the Malay individual**

| Prot Id | Name | Symbol | Alternate Names | Alternate Symbols | Is Genotyped | Is VIP | PD | PK | Has Variant Annotation |
| --- | --- | --- | --- | --- | --- | --- | --- | --- | --- |
| Q92887 | ATP-binding cassette, sub-family C (CFTR/MRP), member 2 | ABCC2 | ATP-BINDING CASSETTE, SUBFAMILY C, MEMBER 2; ABCC2,"ATP-binding cassette sub-family C (CFTR\/MRP) member 2","ATP-binding cassette sub-family C member 2","Human canalicular multispecific organic anion transporter (cMOAT) mRNA, complete cds.","MULTIDRUG RESISTANCE-ASSOCIATED PROTEIN 2; MRP2","MULTISPECIFIC ORGANIC ANION TRANSPORTER, CANALICULAR; CMOAT","OTTHUMP00000020267","canalicular multidrug resistance protein","canalicular multispecific organic anion transporter","canalicular multispecific organic anion transporter 1","multidrug resistance-associated protein 2", | ABC30,"CMOAT","DJS","KIAA1010","MRP2","U63970.1","cMRP", | TRUE | FALSE | PD | PK | TRUE |
| Q7Z442 | polycystic kidney disease 1-like 2 | PKD1L2 | PC1-like 2 protein,"polycystic kidney disease protein 1-like 2","polycystin 1-like 2","polycystin-1L2", | DKFZp686J19100,"FLJ45333","KIAA1879","PC1L2", | FALSE | FALSE | - | - | TRUE |
| Q86SQ4 | G protein-coupled receptor 126 | GPR126 | G-protein coupled receptor 126,"HBV PreS1-transactivated protein 2","OTTHUMP00000040178","developmentally regulated G-protein-coupled receptor","vascular inducible G protein-coupled receptor","vascular-inducible G protein-coupled receptor", | APG1,"DKFZp564D0462","DREG","FLJ14937","PS1TP2","VIGR", | FALSE | FALSE | - | - | TRUE |
| Q6YHU6 | thyroid adenoma associated | THADA | death receptor-interacting protein,"gene inducing thyroid adenomas protein","thyroid adenoma associated gene","thyroid adenoma-associated protein", | FLJ21877,"FLJ44016","FLJ44876","FLJ77530","GITA","KIAA1767", | FALSE | FALSE | - | - | TRUE |
| Q9NVF9 | ethanolamine kinase 2 | ETNK2 | EKI 2,"OTTHUMP00000034314","RP11-74C13.2","ethanolamine kinase-like protein","putative ethanolamine kinase", | EKI2,"FLJ10761","HMFT1716", | TRUE | FALSE | - | - | TRUE |
| Q5VWK5 | interleukin 23 receptor | IL23R | IL-23 receptor,"OTTHUMP00000010660","interleukin-23 receptor", | IL-23R, | FALSE | FALSE | - | - | TRUE |
| Q9Y6C9 | mitochondrial carrier homolog 2 (C. elegans) | MTCH2 | met-induced mitochondrial protein,"mitochondrial carrier 2","mitochondrial carrier homolog 2", | 2310034D24Rik,"HSPC032","MIMP", | FALSE | FALSE | - | - | TRUE |
| Q9Y2K3 | myosin, heavy chain 15 | MYH15 | myosin, heavy polypeptide 15,"myosin-15", | KIAA1000, | FALSE | FALSE | PD | - | TRUE |
| Q7RTV2 | glutathione S-transferase alpha 5 | GSTA5 | GST class-alpha member 5,"OTTHUMP00000016610","glutathione S-transferase A5","glutathione S-transferase A5-5","glutathione transferase A5", |  | TRUE | FALSE | PD | - | FALSE |
| Q8IUL8 | cartilage intermediate layer protein 2 | CILP2 | CILP-2,"cartilage intermediate layer protein-like protein 2", | CLIP-2,"MGC45771", | FALSE | FALSE | - | - | TRUE |
| Q9BXX3 | ankyrin repeat domain 30A | ANKRD30A | OTTHUMP00000019457,"OTTHUMP00000019458","ankyrin repeat domain-containing protein 30A","breast cancer antigen NY-BR-1","serologically defined breast cancer antigen NY-BR-1", | NY-BR-1,"RP11-20F24.1", | FALSE | FALSE | - | - | TRUE |
| Q58EX2 | sidekick homolog 2 (chicken) | SDK2 | Drosophila sidekick-like,"OTTHUMP00000202279","chicken sidekick 2-like","protein sidekick-2","sidekick 2", | FLJ10832,"KIAA1514", | FALSE | FALSE | - | - | TRUE |
| Q3SYG4 | Bardet-Biedl syndrome 9 | BBS9 | Bardet-Biedl syndrome 9,"OTTHUMP00000158833","OTTHUMP00000202918","PTH-responsive osteosarcoma B1 protein","bardet-Biedl syndrome 9 protein","parathyroid hormone-responsive B1","parathyroid hormone-responsive B1 gene protein","protein PTHB1", | PTHB1,"B1","BBS9","C18","D1","MGC118917","PTHB1", | FALSE | FALSE | - | - | TRUE |
| Q96JE7 | SEC16 homolog B (S. cerevisiae) | SEC16B | OTTHUMP00000032954,"RGPR-p117","SEC16 homolog B (S. cerevisiae)","leucine zipper transcription regulator 2","protein SEC16 homolog B","protein transport protein Sec16B","regucalcin gene promoter region-related protein p117","regucalcin gene promotor region related protein", | PGPR-p117,"DKFZp686C2486","FLJ23871","FLJ25761","FLJ33652","FLJ36620","LZTR2","PGPR-p117","RGPR","SEC16B","SEC16S", | FALSE | FALSE | - | - | TRUE |
| Q14CX7 | N(alpha)-acetyltransferase 25, NatB auxiliary subunit | NAA25 | C12orf30,"DKFZp667K2112","FLJ13089","MDM20","N(alpha)-acetyltransferase 25, NatB auxiliary subunit (Non-HGNC Gene)","N-alpha-acetyltransferase 25, NatB auxiliary subunit","N-terminal acetyltransferase B complex subunit NAA25","NAP1","mitochondrial distribution and morphology 20","natB complex subunit MDM20", |  | FALSE | FALSE | - | - | TRUE |
| Q9Y2K2 | SIK family kinase 3 | SIK3 | FLJ12240,"KIAA0999","OTTHUMP00000069593","OTTHUMP00000069594","OTTHUMP00000198139","QSK","SIK family kinase 3 (Non-HGNC Gene)","SIK-3","salt-inducible kinase 3","serine/threonine-protein kinase QSK","serine/threonine-protein kinase SIK3", | KIAA0999," L19"," QSK", | FALSE | FALSE | - | - | TRUE |
| O14513 | NCK-associated protein 5 | NCKAP5 | ERIH1,"ERIH2","FLJ34870","NAP-5","NAP5","NCK-associated protein 5 (Non-HGNC Gene)","Nck associated protein 5","OTTHUMP00000204126","OTTHUMP00000204279","nck-associated protein 5","peripheral clock protein 2", | ERIH1," ERIH2", | FALSE | FALSE | - | - | TRUE |
| P34947 | G protein-coupled receptor kinase 5 | GRK5 | g protein-coupled receptor kinase GRK5, | GPRK5, | TRUE | FALSE | PD | - | TRUE |
| Q96J66 | ATP-binding cassette, sub-family C (CFTR/MRP), member 11 | ABCC11 | ATP-binding cassette protein C11,"ATP-binding cassette sub-family C member 11","ATP-binding cassette transporter MRP8","ATP-binding cassette transporter sub-family C member 11","ATP-binding cassette, sub-family C, member 11","OTTHUMP00000164191","multi-resistance protein 8","multidrug resistance-associated protein 8", | ABCC11,"EWWD","MRP8","WW", | TRUE | FALSE | - | PK | FALSE |
| Q9H221 | ATP-binding cassette, sub-family G (WHITE), member 8 | ABCG8 | ATP-binding cassette sub-family G member 8,"ATP-binding cassette, sub-family G (WHITE), member 8 (sterolin 2)","ATP-binding cassette, subfamily G, member 8","sterolin 2","sterolin-2", | ABCG8,"GBD4","MGC142217","STSL", | FALSE | FALSE | PD | - | TRUE |
| P16442 | ABO blood group (transferase A, alpha 1-3-N-acetylgalactosaminyltransferase; transferase B, alpha 1-3-galactosyltransferase) | ABO | ABO blood group,"ABO glycocyltransferase","ABO glycosyltransferase","B(A) alpha-1,3-galactosyltransferase","alpha 1-3-N-acetylgalactosaminyltransferase","fucosylglycoprotein 3-alpha-galactosyltransferase","fucosylglycoprotein alpha-N-acetylgalactosaminyltransferase","glycoprotein-fucosylgalactoside alpha-N-acetylgalactosaminyltransferase","glycoprotein-fucosylgalactoside alpha-galactosyltransferase","histo-blood group A transferase","histo-blood group A2 transferase","histo-blood group ABO protein","histo-blood group ABO system transferase","histo-blood group B transferase", | A3GALNT,"A3GALT1","GTB","NAGAT", | FALSE | FALSE | PD | - | TRUE |
| O00763 | acetyl-CoA carboxylase beta | ACACB | ACC-beta,"acetyl-CoA carboxylase 2","acetyl-Coenzyme A carboxylase beta", | ACC2,"ACCB","HACC275", | FALSE | FALSE | PD | - | FALSE |
| P42330 | aldo-keto reductase family 1, member C3 (3-alpha hydroxysteroid dehydrogenase, type II) | AKR1C3 | 3-alpha hydroxysteroid dehydrogenase type IIb,"3-alpha-HSD type II, brain","OTTHUMP00000018996","aldo-keto reductase family 1 member C3","aldo-keto reductase family 1, member C3","chlordecone reductase","chlordecone reductase homolog","chlordecone reductase homolog HAKRb","dihydrodiol dehydrogenase 3","dihydrodiol dehydrogenase X","hydroxysteroid (17-beta) dehydrogenase 5","indanol dehydrogenase","prostaglandin F synthase","testosterone 17-beta-dehydrogenase 5","trans-1,2-dihydrobenzene-1,2-diol dehydrogenase","type II 3a-hydroxysteroid dehydrogenase","type IIb 3-alpha hydroxysteroid dehydrogenase", | DD3,"DDX","HA1753","HAKRB","HAKRe","HSD17B5","KIAA0119","PGFS","hluPGFS", | FALSE | FALSE | PD | PK | FALSE |
| P50995 | annexin A11 | ANXA11 | 56 kDa autoantigen,"CAP-50","OTTHUMP00000019955","OTTHUMP00000019956","OTTHUMP00000019957","OTTHUMP00000019958","OTTHUMP00000059806","annexin XI","annexin-11","autoantigen, 56-kD","calcyclin-associated annexin 50", | ANX11,"CAP50", | FALSE | FALSE | PD | - | TRUE |
| P61769 | beta-2-microglobulin | B2M | beta chain of MHC class I molecules,"beta-2-microglobin", |  | FALSE | FALSE | PD | PK | FALSE |
| P38398 | breast cancer 1, early onset | BRCA1 | BRCA1/BRCA2-containing complex, subunit 1,"OTTHUMP00000212147","OTTHUMP00000212148","OTTHUMP00000212149","OTTHUMP00000212150","OTTHUMP00000212151","OTTHUMP00000212155","RING finger protein 53","breast and ovarian cancer susceptibility protein 1","breast and ovarian cancer sususceptibility protein","breast cancer type 1 susceptibility protein","breast-ovarian cancer, included", | BRCAI,"BRCC1","BROVCA1","IRIS","PNCA4","PSCP","RNF53", | FALSE | TRUE | PD | - | TRUE |
| P51587 | breast cancer 2, early onset | BRCA2 | BRCA1/BRCA2-containing complex, subunit 2,"Fanconi anemia, complementation group D1","OTTHUMP00000018803","OTTHUMP00000042401","breast and ovarian cancer susceptibility gene, early onset","breast cancer 2 tumor suppressor","breast cancer susceptibility protein BRCA2","breast cancer type 2 susceptibility protein","fanconi anemia group D1 protein", | BRCC2,"BROVCA2","FACD","FAD","FAD1","FANCB","FANCD","FANCD1","GLM3","PNCA2", | FALSE | FALSE | PD | - | FALSE |
| O43916 | carbohydrate (keratan sulfate Gal-6) sulfotransferase 1 | CHST1 | GST-1,"KSST","carbohydrate (chondroitin 6/keratan) sulfotransferase 1","carbohydrate sulfotransferase 1","galactose/N-acetylglucosamine/N-acetylglucosamine 6-O-sulfotransferase 1","keratan sulfate Gal-6 sulfotransferase", | C6ST,"KS6ST","KSGAL6ST","KSGal6ST", | TRUE | FALSE | PD | - | FALSE |
| P78329 | cytochrome P450, family 4, subfamily F, polypeptide 2 | CYP4F2 | CYPIVF2,"cytochrome P450 4F2","cytochrome P450, subfamily IVF, polypeptide 2","cytochrome P450-LTB-omega","leukotriene B4 omega-hydroxylase","leukotriene-B(4) 20-monooxygenase 1","leukotriene-B(4) omega-hydroxylase 1","leukotriene-B4 20-monooxygenase", | CPF2, | TRUE | FALSE | PD | PK | TRUE |
| Q8TE73 | dynein, axonemal, heavy chain 5 | DNAH5 | axonemal beta dynein heavy chain 5,"ciliary dynein heavy chain 5","dynein heavy chain 5","dynein heavy chain 5, axonemal","dynein, axonemal, heavy polypeptide 5", | CILD3,"DNAHC5","Dnahc5","FLJ46759","HL1","KIAA1603","KTGNR","PCD", | FALSE | FALSE | - | - | TRUE |
| P36888 | fms-related tyrosine kinase 3 | FLT3 | CD135 antigen,"FL cytokine receptor","FLT-3","FLT3 receptor tyrosine kinase","OTTHUMP00000042340","OTTHUMP00000214829","STK-1","fetal liver kinase 2","fms-like tyrosine kinase 3","growth factor receptor tyrosine kinase type III","stem cell tyrosine kinase 1","tyrosine-protein kinase receptor FLT3", | CD135,"FLK2","STK1", | FALSE | FALSE | PD | - | FALSE |
| O14976 | cyclin G associated kinase | GAK | OTTHUMP00000217314,"cyclin-G-associated kinase", | FLJ16629,"FLJ40395","MGC99654", | FALSE | FALSE | - | - | TRUE |
| P10912 | growth hormone receptor | GHR | GH receptor,"growth hormone binding protein","serum binding protein","somatotropin receptor", | GHBP, | FALSE | FALSE | PD | - | TRUE |
| Q9Y5Y4 | G protein-coupled receptor 44 | GPR44 | chemoattractant receptor homologous molecule expressed on T helper type 2 cells,"chemoattractant receptor-homologous molecule expressed on TH2 cells","putative G-protein coupled receptor 44", | CD294,"CRTH2","DL1R","DP2", | FALSE | FALSE | PD | - | FALSE |
| P11717 | insulin-like growth factor 2 receptor | IGF2R | 300 kDa mannose 6-phosphate receptor,"CI Man-6-P receptor","CI-MPR","IGF-II receptor","Insulin-like growth factor-2 receptor (mannose-6-phosphate receptor, cation-independent)","M6P/IGF2 receptor","M6P/IGF2R","M6PR","MPR 300","OTTHUMP00000017536","cation-independent mannose-6 phosphate receptor","cation-independent mannose-6-phosphate receptor","insulin-like growth factor II receptor", | CD222,"CIMPR","M6P-R","MPR1","MPRI", | FALSE | FALSE | - | - | TRUE |
| P27930 | interleukin 1 receptor, type II | IL1R2 | CD121 antigen-like family member B,"CDw121b","IL-1R-2","IL-1R-beta","IL-1RT-2","IL-1RT2","Interleukin-1 receptor, beta","OTTHUMP00000207969","antigen CDw121b","interleukin-1 receptor beta","interleukin-1 receptor type 2","interleukin-1 receptor type II","type II interleukin-1 receptor, beta","type II receptor", | CD121b,"IL1RB","MGC47725", | FALSE | FALSE | - | PK | FALSE |
| O14896 | interferon regulatory factor 6 | IRF6 | IRF-6,"OTTHUMP00000034677","OTTHUMP00000034678","Popliteala pterygium syndrome", | LPS,"OFC6","PIT","PPS","VWS", | FALSE | FALSE | - | - | TRUE |
| P20702 | integrin, alpha X (complement component 3 receptor 4 subunit) | ITGAX | CD11 antigen-like family member C,"OTTHUMP00000163299","integrin alpha X","integrin alpha-X","integrin, alpha X (antigen CD11C (p150), alpha polypeptide)","leu M5, alpha subunit","leukocyte adhesion glycoprotein p150,95 alpha chain","leukocyte adhesion receptor p150,95","leukocyte surface antigen p150,95, alpha subunit","myeloid membrane antigen, alpha subunit","p150 95 integrin alpha chain", | CD11C,"CD11c","SLEB6", | FALSE | FALSE | PD | - | TRUE |
| P05107 | integrin, beta 2 (complement component 3 receptor 3 and 4 subunit) | ITGB2 | Integrin, beta-2 (antigen CD18 (p95), lymphocyte function-associated,"OTTHUMP00000115278","OTTHUMP00000115279","OTTHUMP00000115281","OTTHUMP00000115282","cell surface adhesion glycoprotein (LFA-1/CR3/P150,959 beta subunit precursor)","cell surface adhesion glycoprotein LFA-1/CR3/P150,959 beta subunit precursor)","cell surface adhesion glycoproteins LFA-1/CR3/p150,95 subunit beta","complement receptor C3 beta-subunit","complement receptor C3 subunit beta","integrin beta chain, beta 2","integrin beta-2","integrin, beta 2","integrin, beta 2 (antigen CD18 (p95), lymphocyte function-associated antigen 1; macrophage antigen 1 (mac-1) beta subunit)","leukocyte cell adhesion molecule CD18","leukocyte-associated antigens CD18/11A, CD18/11B, CD18/11C", | CD18,"LAD","LCAMB","LFA-1","MAC-1","MF17","MFI7", | FALSE | FALSE | PD | - | FALSE |
| P07942 | laminin, beta 1 | LAMB1 | OTTHUMP00000195576,"cutis laxa with marfanoid phenotype","laminin B1 chain","laminin subunit beta-1","laminin-1 subunit beta","laminin-10 subunit beta","laminin-12 subunit beta","laminin-2 subunit beta","laminin-6 subunit beta","laminin-8 subunit beta", | CLM,"MGC142015", | FALSE | FALSE | - | - | TRUE |
| P18858 | ligase I, DNA, ATP-dependent | LIG1 | DNA ligase 1,"DNA ligase I","polydeoxyribonucleotide synthase ATP 1","polydeoxyribonucleotide synthase [ATP] 1", | MGC117397,"MGC130025", | FALSE | FALSE | PD | - | FALSE |
| P49917 | ligase IV, DNA, ATP-dependent | LIG4 | DNA joinase,"DNA ligase 4","DNA ligase IV","DNA repair enzyme","Sealase","polydeoxyribonucleotide synthase","polydeoxyribonucleotide synthase [ATP] 4","polynucleotide ligase","sealase", |  | FALSE | FALSE | - | - | TRUE |
| Q08397 | lysyl oxidase-like 1 | LOXL1 | lysyl oxidase homolog 1,"lysyl oxidase-like protein 1", | LOL,"LOXL", | FALSE | FALSE | - | - | TRUE |
| Q04671 | oculocutaneous albinism II | OCA2 | P protein,"eye color 2 (central brown)","eye color 3 (brown)","hair color 3 (brown)","melanocyte-specific transporter protein","oculocutaneous albinism II (pink-eye dilution (murine) homolog)","oculocutaneous albinism II (pink-eye dilution homolog, mouse)","pink-eyed dilution protein homolog","total brown iris pigmentation", | BEY,"BEY1","BEY2","BOCA","D15S12","EYCL","EYCL2","EYCL3","HCL3","P","PED","SHEP1", | FALSE | FALSE | - | - | TRUE |
| Q9BZA7 | protocadherin 11 X-linked | PCDH11X | OTTHUMP00000023640,"OTTHUMP00000023641","OTTHUMP00000023642","OTTHUMP00000023643","OTTHUMP00000217565","protocadherin 11","protocadherin 11X","protocadherin X","protocadherin on the X chromosome","protocadherin-11 X-linked","protocadherin-S", | PCDH-X,"PCDH11","PCDHX","PCDHY", | FALSE | FALSE | - | - | TRUE |
| O60658 | phosphodiesterase 8A | PDE8A | OTTHUMP00000192898,"cAMP-specific cyclic nucleotide phosphodiesterase 8A","high affinity cAMP-specific and IBMX-insensitive 3',5'-cyclic phosphodiesterase 8A","high-affinity cAMP-specific and IBMX-insensitive 3',5'-cyclic phosphodiesterase 8A", | FLJ16150,"HsT19550", | FALSE | FALSE | PD | - | FALSE |
| O60437 | periplakin | PPL | 190 kDa paraneoplastic pemphigus antigen,"195 kDa cornified envelope","195 kDa cornified envelope precursor protein","OTTHUMP00000159922", | KIAA0568,"MGC134872", | FALSE | FALSE | PD | - | FALSE |
| Q9UGJ0 | protein kinase, AMP-activated, gamma 2 non-catalytic subunit | PRKAG2 | 5'-AMP-activated protein kinase subunit gamma-2,"AMP-activated protein kinase gamma2 subunit","AMPK gamma2","AMPK subunit gamma-2","H91620p protein","OTTHUMP00000211956", | AAKG,"AAKG2","CMH6","H91620P","H91620p","WPWS", | FALSE | FALSE | PD | - | TRUE |
| Q7Z5J4 | retinoic acid induced 1 | RAI1 | OTTHUMP00000065594,"Smith-Magenis syndrome chromosome region","retinoic acid-induced protein 1", | DKFZP434A139,"DKFZp434A139","KIAA1820","MGC12824","SMCR","SMS", | FALSE | FALSE | - | - | TRUE |
| P21817 | ryanodine receptor 1 (skeletal) | RYR1 | RYR-1,"central core disease of muscle","ryanodine receptor 1","ryanodine receptor type1","sarcoplasmic reticulum calcium release channel","skeletal muscle calcium release channel","skeletal muscle ryanodine receptor","skeletal muscle-type ryanodine receptor","type 1-like ryanodine receptor", | CCO,"MHS","MHS1","RYDR","RYR","SKRR", | FALSE | FALSE | PD | - | TRUE |
| Q9NZJ4 | spastic ataxia of Charlevoix-Saguenay (sacsin) | SACS | OTTHUMP00000018109,"OTTHUMP00000018111","dnaJ homolog subfamily C member 29","sacsin", | ARSACS,"DKFZp686B15167","DNAJC29","KIAA0730", | FALSE | FALSE | PD | - | FALSE |
| O60243 | heparan sulfate 6-O-sulfotransferase 1 | HS6ST1 | HS6ST-1,"heparan-sulfate 6-O-sulfotransferase 1","heparan-sulfate 6-sulfotransferase", | DKFZp547H098,"FLJ25392","HS6ST","MGC116899","MGC116901", | FALSE | FALSE | - | - | TRUE |
| O95425 | supervillin | SVIL | OTTHUMP00000019389,"archvillin","membrane-associated F-actin binding protein p205","p205/p250", | DKFZp686A17191, | FALSE | FALSE | - | - | TRUE |
| O15455 | toll-like receptor 3 | TLR3 |  | CD283, | FALSE | FALSE | - | - | TRUE |
| Q02880 | topoisomerase (DNA) II beta 180kDa | TOP2B | DNA topoisomerase 2-beta,"DNA topoisomerase II beta","DNA topoisomerase II, 180 kD","DNA topoisomerase II, beta isozyme","OTTHUMP00000208318","U937 associated antigen","antigen MLAA-44","topo II beta","topoisomerase (DNA) II beta (180kD)","topoisomerase II beta","topoisomerase IIb", | TOPIIB,"top2beta", | FALSE | FALSE | PD | - | FALSE |
| P20396 | thyrotropin-releasing hormone | TRH | OTTHUMP00000216040,"prothyroliberin", | MGC125964,"MGC125965", | FALSE | FALSE | PD | - | FALSE |
| P57075 | ubiquitin associated and SH3 domain containing A | UBASH3A | OTTHUMP00000109368,"SH3","T-cell ubiquitin ligand protein","UBA","cbl-interacting protein 4","gene similar to UBA containing SH3 domain","suppressor of T-cell receptor signaling 2","ubiquitin associated and SH3 domain containing, A","ubiquitin-associated and SH3 domain-containing protein A", | CLIP4,"STS-2","TULA", | FALSE | FALSE | - | - | TRUE |
| O75445 | Usher syndrome 2A (autosomal recessive, mild) | USH2A | OTTHUMP00000035145,"OTTHUMP00000063383","Usherin","usher syndrome type IIa protein","usher syndrome type-2A protein","usherin", | RP39,"US2","USH2","dJ1111A8.1", | FALSE | FALSE | - | - | TRUE |
| P04275 | von Willebrand factor | VWF | Coagulation factor VIII VWF (von Willebrand factor),"coagulation factor VIII VWF", | F8VWF,"VWD", | FALSE | FALSE | PD | - | FALSE |
| Q9Y4H2 | insulin receptor substrate 2 | IRS2 | OTTHUMP00000018696, | IRS-2, | FALSE | FALSE | PD | - | FALSE |
| Q9NZL3 | zinc finger protein 224 | ZNF224 | bone marrow zinc finger 2,"zinc finger 2, bone marrow","zinc finger protein 233","zinc finger protein 255","zinc finger protein 27","zinc finger protein 27 (KOX 22)","zinc finger protein KOX22","zinc finger protein ZNF255", | BMZF-2,"BMZF2","KOX22","ZNF233","ZNF255","ZNF27", | FALSE | FALSE | - | - | TRUE |
| Q96JP5 | zinc finger protein 91 homolog (mouse) | ZFP91 | OTTHUMP00000196612,"OTTHUMP00000196613","zfp-91","zinc finger protein 91","zinc finger protein 91 homolog","zinc finger protein homologous to Zfp91 in mouse", | FKSG11,"PZF","ZNF757", | FALSE | FALSE | - | - | TRUE |
| Q9NNW7 | thioredoxin reductase 2 | TXNRD2 | OTTHUMP00000195708,"selenoprotein Z","thioredoxin reductase 2, mitochondrial","thioredoxin reductase 3","thioredoxin reductase TR3","thioredoxin reductase beta", | SELZ,"TR","TR-BETA","TR3","TRXR2", | TRUE | FALSE | - | - | TRUE |
| Q9NST1 | patatin-like phospholipase domain containing 3 | PNPLA3 | OTTHUMP00000028602,"PNPLA3","acylglycerol O-acyltransferase","calcium-independent phospholipase A2-epsilon","chromosome 22 open reading frame 20","hypothetical protein dJ796I17.1","iPLA2-epsilon","patatin-like phospholipase domain-containing protein 3", | ADPN,"C22orf20","FLJ22012","adiponutrin","dJ796I17.1","iPLA(2)epsilon", | FALSE | FALSE | - | - | TRUE |
| O43868 | solute carrier family 28 (sodium-coupled nucleoside transporter), member 2 | SLC28A2 | CNT 2,"CONCENTRATIVE NUCLEOSIDE TRANSPORTER 2; CNT2","Human Na+-dependent purine specific transporter mRNA, complete cds.","Na(+)/nucleoside cotransporter 2","SODIUM-DEPENDENT PURINE NUCLEOSIDE TRANSPORTER 1; SPNT1","SOLUTE CARRIER FAMILY 28, MEMBER 2; SLC28A2","SPNT","concentrative nucleoside transporter 2","sodium-coupled nucleoside transporter 2","sodium/nucleoside cotransporter 2","sodium/purine nucleoside co-transporter","solute carrier family 28 member 2", | CNT2,"FLJ21468","HCNT2","HsT17153","MGC138252","SPNT1","U84392.1", | TRUE | FALSE | PD | PK | TRUE |
| O00337 | solute carrier family 28 (sodium-coupled nucleoside transporter), member 1 | SLC28A1 | CNT 1,"CONCENTRATIVE NUCLEOSIDE TRANSPORTER 1; CNT1","Human Na+/nucleoside cotransporter (hCNT1c) mRNA, complete cds.","Na(+)/nucleoside cotransporter 1","OTTHUMP00000192893","SOLUTE CARRIER FAMILY 28, MEMBER 1; SLC28A1","concentrative nucleoside transporter 1","sodium-coupled nucleoside transporter 1","sodium/nucleoside cotransporter 1","solute carrier family 28 member 1", | CNT1,"HCNT1","SLC28A1","U62968.1", | TRUE | FALSE | PD | PK | TRUE |
| P02671 | fibrinogen alpha chain | FGA | fibrinogen, A alpha polypeptide,"fibrinogen, alpha chain, isoform alpha preproprotein","fibrinogen, alpha polypeptide", | Fib2,"MGC119422","MGC119423","MGC119425", | TRUE | FALSE | PD | - | FALSE |
| P04114 | apolipoprotein B (including Ag(x) antigen) | APOB | OTTHUMP00000115994,"apo B-100","apoB-100","apoB-48","apolipoprotein B","apolipoprotein B-100","apolipoprotein B48", | FLDB,"LDLCQ4", | TRUE | FALSE | PD | - | TRUE |
