# Supplementary material for: Systematic Pharmacogenomics Analysis of a Malay Whole Genome: Proof of Concept for Personalized Medicine
Source: PLoS One. 2013 Aug 23;8(8):e71554. doi: 10.1371/journal.pone.0071554 (PMC3751891; doi:10.1371/journal.pone.0071554)
Supplement: Table S7 — Risk alleles and corresponding risk magnitude and odds ratio. (DOCX) [file pone.0071554.s010.docx]

**Table S7: Risk alleles and corresponding risk magnitude and odds ratio**

| Disease | Risk allele | Magnitude | Odds ratio |
| --- | --- | --- | --- |
| LC | rs1517114 | 0 | 4.1 |
| LC | rs4233356 | 0 | 2.51 |
| LC | rs12000445 | 0 | 1.49 |
| LC | rs2352028 | 2 | 1.46 |
| LC | rs969088 | 0 | 1.43 |
| LC | rs2736100 | 1.3 | 1.38 |
| LC | rs1878022 | 0 | 1.33 |
| LC | rs9981861 | 0 | 1.33 |
| LC | rs3850370 | 0 | 1.31 |
| LC | rs2131877 | 0 | 1.3 |
| LC | rs36600 | 0 | 1.29 |
| LC | rs2808630 | 0 | 1.22 |
| LC | rs9387478 | 0 | 1.18 |
| LC | rs753955 | 0 | 1.18 |
| LC | rs402710 | 0 | 1.18 |
| LC | rs748404 | 1.25 | 1.15 |
| LC | rs4975616 | 0 | 1.15 |
| LC | rs401681 | 0 | 1.15 |
| LC | rs10197940 | 0 | 1.12 |
| LC | rs4254535 | 0 | 1.12 |
| PC | rs10861905 | 0 | 33.95 |
| PC | rs5971305 | 0 | 33.69 |
| PC | rs5965182 | 0 | 11.73 |
| PC | rs1527243 | 0 | 6.67 |
| PC | rs10210358 | 0 | 5.34 |
| PC | rs11122834 | 0 | 4.93 |
| PC | rs17779457 | 0 | 2.4 |
| PC | rs4242384 | 0 | 1.88 |
| PC | rs6983561 | 0 | 1.87 |
| PC | rs16901979 | 0 | 1.8 |
| PC | rs10505483 | 0 | 1.73 |
| PC | rs10090154 | 2.1 | 1.68 |
| PC | rs4242382 | 0 | 1.66 |
| PC | rs1447295 | 0 | 1.6 |
| PC | rs1002979 | 0 | 1.59 |
| PC | rs4463179 | 0 | 1.56 |
| PC | rs7789197 | 0 | 1.52 |
| PC | rs5751168 | 0 | 1.44 |
| PC | rs784411 | 0 | 1.42 |
| PC | rs6983267 | 0 | 1.42 |
| PC | rs3789080 | 0 | 1.41 |
| PC | rs7501939 | 0 | 1.41 |
| PC | rs817826 | 0 | 1.41 |
| PC | rs12682851 | 0 | 1.39 |
| PC | rs12653946 | 0 | 1.39 |
| PC | rs1016343 | 0 | 1.37 |
| PC | rs7694725 | 0 | 1.36 |
| PC | rs10277209 | 0 | 1.36 |
| PC | rs6089829 | 0 | 1.35 |
| PC | rs1512268 | 0 | 1.35 |
| PC | rs1243647 | 0 | 1.33 |
| PC | rs735172 | 0 | 1.31 |
| PC | rs6763848 | 0 | 1.3 |
| PC | rs6507016 | 0 | 1.28 |
| PC | rs103294 | 0 | 1.28 |
| PC | rs1859962 | 0 | 1.27 |
| PC | rs10993994 | 2 | 1.25 |
| PC | rs9757252 | 0 | 1.25 |
| PC | rs9351730 | 0 | 1.25 |
| PC | rs543686 | 0 | 1.24 |
| PC | rs5945572 | 0 | 1.23 |
| PC | rs1866967 | 0 | 1.22 |
| PC | rs445114 | 0 | 1.22 |
| PC | rs339331 | 0 | 1.22 |
| PC | rs7127900 | 0 | 1.22 |
| PC | rs2735839 | 0 | 1.2 |
| PC | rs5945619 | 0 | 1.19 |
| PC | rs9600079 | 0 | 1.18 |
| PC | rs2660753 | 0 | 1.18 |
| PC | rs9364554 | 0 | 1.17 |
| PC | rs742134 | 0 | 1.16 |
| PC | rs5759167 | 0 | 1.16 |
| PC | rs13385191 | 0 | 1.15 |
| PC | rs1983891 | 0 | 1.15 |
| PC | rs8102476 | 0 | 1.12 |
| PC | rs10934853 | 0 | 1.12 |
| PC | rs10486567 | 0 | 1.12 |
| PC | rs11672691 | 0 | 1.11 |
| PC | rs10936632 | 0 | 1.11 |
| PC | rs17021918 | 0 | 1.11 |
| PC | rs7679673 | 0 | 1.1 |
| PC | rs12500426 | 0 | 1.08 |
| PC | rs10875943 | 0 | 1.07 |
| PC | rs7629490 | 0 | 1.06 |
| PC | rs5919432 | 0 | 1.06 |
| PC | rs2121875 | 0 | 1.05 |
| PC | rs12155172 | 0 | 1.05 |
| SCHIZ | rs16977195 | 0 | 6.01 |
| SCHIZ | rs7902091 | 0 | 5.33 |
| SCHIZ | rs4846033 | 0 | 2.87 |
| SCHIZ | rs151222 | 0 | 2.1 |
| SCHIZ | rs10911902 | 0 | 1.79 |
| SCHIZ | rs10429924 | 0 | 1.61 |
| SCHIZ | rs643410 | 0 | 1.6 |
| SCHIZ | rs11265461 | 0 | 1.45 |
| SCHIZ | rs12611334 | 0 | 1.4 |
| SCHIZ | rs2048485 | 0 | 1.4 |
| SCHIZ | rs1170612 | 0 | 1.4 |
| SCHIZ | rs12966547 | 0 | 1.4 |
| SCHIZ | rs4765905 | 0 | 1.36 |
| SCHIZ | rs2159767 | 0 | 1.33 |
| SCHIZ | rs7930295 | 0 | 1.3 |
| SCHIZ | rs12140439 | 0 | 1.3 |
| SCHIZ | rs583583 | 0 | 1.3 |
| SCHIZ | rs2726807 | 0 | 1.29 |
| SCHIZ | rs11038167 | 0 | 1.29 |
| SCHIZ | rs1635 | 0 | 1.28 |
| SCHIZ | rs9611198 | 0 | 1.22 |
| SCHIZ | rs7914558 | 0 | 1.22 |
| SCHIZ | rs7897654 | 0 | 1.2 |
| SCHIZ | rs589249 | 0 | 1.2 |
| SCHIZ | rs12922317 | 0 | 1.17 |
| SCHIZ | rs7045881 | 0 | 1.16 |
| SCHIZ | rs10761482 | 0 | 1.16 |
| SCHIZ | rs16915157 | 0 | 1.13 |
| SCHIZ | rs433598 | 0 | 1.13 |
| SCHIZ | rs1198588 | 0 | 1.12 |
| SCHIZ | rs1625579 | 0 | 1.12 |
| SCHIZ | rs7192086 | 0 | 1.12 |
| SCHIZ | rs7709645 | 0 | 1.11 |
| SCHIZ | rs10894294 | 0 | 1.1 |
| SCHIZ | rs10503256 | 0 | 1.1 |
| SCHIZ | rs11220082 | 0 | 1.1 |
| SCHIZ | rs2239547 | 0 | 1.1 |
| SCHIZ | rs548181 | 0 | 1.1 |
| SCHIZ | rs11130874 | 0 | 1.1 |
| SCHIZ | rs6703335 | 0 | 1.09 |
| SCHIZ | rs1009080 | 0 | 1.09 |
| SCHIZ | rs2312147 | 0 | 1.09 |
| SCHIZ | rs1502844 | 0 | 1.09 |
| SCHIZ | rs12699131 | 0 | 1.08 |
| SCHIZ | rs2252865 | 0 | 1.08 |
| SCHIZ | rs1869901 | 0 | 1.07 |
| SCHIZ | rs489332 | 0 | 0.11 |
| SCHIZ | rs1920592 | 0 | 0.11 |
| SCHIZ | rs2323266 | 0 | 0.1 |
| SCHIZ | rs1351267 | 0 | 0.09 |
